# Design and Evaluation of a Source-Grounded Medical LLM for Clinical Decision Support and Patient Care in Trustworthy Diagnostic Systems

**DOI:** 10.3390/diagnostics16193142

**Published:** 2026-09-27

**Authors:** Muhammad Jamil, Adnan Kavak, Sevinç İlhan Omurca, Hossein Fotouhi

**Affiliations:** 1 Department of Computer Engineering, Kocaeli University, Kocaeli 41001, Türkiye; jamil138.amin@gmail.com (M.J.); silhan@kocaeli.edu.tr (S.İ.O.); 2 Wireless Information and Intelligent Systems (WINS) Research Center, Kocaeli University, Kocaeli 41001, Türkiye; 3 Department of Computer Science and Engineering, Mälardalen University (MDU), 72123 Västerås, Sweden

**Keywords:** medical large language model, diagnostic decision support, clinical diagnosis, retrieval-augmented generation, medical artificial intelligence, clinical decision support

## Abstract

**Background/Objectives:** Large language models (LLMs) are increasingly explored for clinical decision support and digital health applications. However, reliable diagnostic assistance remains challenging for low-resource medical languages such as Turkish due to limited source grounding, transparency, clinical safety, and localized medical knowledge. This study presents TurkishMedLLM, a source-grounded and safety-aware Turkish medical LLM designed for clinician-supervised diagnostic decision support, symptom interpretation, and patient care. **Methods:** The methodology integrates multi-source Turkish medical data ingestion, schema standardization, duplicate removal, quality filtering, supervised fine-tuning, embedding generation, vector indexing, Qwen3-8B fine-tuning, retrieval-augmented generation (RAG), and multi-layer evaluation. The system was evaluated using retrieval metrics, ROUGE, RAGAS, DeepEval, and clinical safety assessments. As a use case, TurkishMedLLM was integrated into the AI-based Diabetes Care (AIDCare) mHealth platform, which supports patient queries related to lifestyle management, symptoms, diagnosis, and treatment of diabetes. The system generates safety-aware responses with clinician-in-the-loop validation before delivery through the mobile application. **Results:** After pre-processing, the final dataset comprised 232,926 unique documents, including 210,791 Turkish medical question–answer pairs and 22,135 hospital medical articles. The retrieval module achieved Hit Rate@1, Hit Rate@3, and Hit Rate@5 of 94.67%, 98.67%, and 100.00%, respectively, indicating consistent retrieval of clinically relevant evidence. QLoRA fine-tuning achieved a validation loss of 0.9373 and ROUGE-1, ROUGE-2, ROUGE-L, and ROUGE-Lsum scores of 0.8338, 0.5476, 0.7836, and 0.7372, respectively. The fine-tuned RAG system achieved RAGAS faithfulness and response relevancy scores of 0.91 and 0.88, respectively, while DeepEval achieved an answer relevancy score of 0.90. Clinical safety assessment achieved a caution score of 0.93, indicating generally evidence-grounded and clinically cautious responses for symptom interpretation and diagnosis-related patient support. **Conclusions:** Combining retrieval grounding, parameter-efficient fine-tuning, and multi-layer safety evaluation provides a promising approach for clinician-supervised medical AI in Turkish. TurkishMedLLM demonstrates potential for symptom interpretation, differential diagnostic support, and trustworthy digital health applications.

## 1. Introduction

In today’s digital health landscape, artificial intelligence (AI) is transforming how medical data are accessed, interpreted, and utilized to support clinical decision making and medical diagnosis [1,2]. With the rapid advancement of large language models (LLMs), new opportunities have emerged for clinician-assisted diagnostic decision support, symptom interpretation, patient education, clinical documentation, and decision-support workflows [3,4]. These models can understand natural language descriptions of symptoms, medications, chronic diseases, and lifestyle factors, enabling more effective communication between patients and healthcare professionals. However, healthcare is not a conventional text-generation domain. Medical responses must be accurate, evidence-based, clinically cautious, source-grounded, and understandable because they may influence diagnostic reasoning, patient management, and subsequent clinical decisions [5,6].

The central problem addressed in this study is the development of a trustworthy Turkish medical large language model (LLM) capable of providing source-grounded and clinically cautious support for medical question answering, symptom interpretation, patient education, and clinician-supervised diagnostic decision support. Although LLMs provide strong natural-language generation capabilities, their application to medical contexts requires additional mechanisms for retrieving relevant evidence, maintaining traceability to information sources, communicating uncertainty, and avoiding unsupported diagnostic or treatment recommendations [7]. These requirements are particularly important when generated responses concern potentially serious symptoms or ambiguous clinical conditions. Accordingly, this study formulates medical response generation as a source-grounded diagnostic support task in which retrieval relevance, factual faithfulness, clinical caution, and risk-aware response behavior are jointly considered. The formal mathematical formulation of this task is presented in Section 3.1.

Medical language generation can directly influence how users understand and act upon medical information [8]. A response that is fluent but insufficiently supported by reliable evidence may communicate an inappropriate level of certainty and may not adequately reflect the limitations of the available information. In medical settings, therefore, response quality cannot be characterized solely by linguistic fluency. A medical LLM should be able to retrieve clinically relevant evidence, maintain source traceability, communicate diagnostic uncertainty when the evidence is insufficient, avoid presenting unsupported definitive diagnoses, and provide appropriate guidance to seek professional medical assessment when potentially serious or ambiguous conditions are identified [7]. These requirements motivate an architecture in which evidence retrieval, model adaptation, response generation, and safety-oriented controls are considered as complementary components rather than as independent text-generation capabilities.

In this study, patient education and clinical decision support are treated as distinct but complementary use cases. Patient education refers to the provision of understandable, source-grounded information about symptoms, diseases, medications, prevention, and general health topics without replacing professional medical assessment. In contrast, clinical decision support refers to the use of retrieved medical evidence and clinically cautious model-generated information to assist qualified healthcare professionals in assessment and decision-making. TurkishMedLLM is designed to support both settings while maintaining a clear safety boundary: it is not intended to function as an autonomous diagnostic or treatment system. In clinical decision-support scenarios, the system operates within a clinician-supervised workflow, where source grounding, uncertainty communication, safety-aware response control, and appropriate escalation guidance are used to support professional judgment and reduce the risk of unsupported medical recommendations.

Another important consideration is the linguistic and clinical reliability of medical LLMs in low-resource languages such as Turkish. The availability of localized medical resources, annotated datasets, and evaluation benchmarks for Turkish remains comparatively limited [9]. This creates challenges for adapting general-purpose multilingual LLMs to Turkish medical terminology, patient-oriented language, and domain-specific clinical queries. In addition, medical LLM evaluation requires more than conventional language-generation measures because response quality depends on multiple dimensions, including evidence retrieval, factual faithfulness, relevance, language quality, clinical caution, and safety behavior. Human-in-the-loop assessment can therefore provide an important complementary perspective by examining whether generated responses are consistent with clinical expectations and appropriately positioned as support for, rather than replacement of, professional medical judgment [10]. These considerations motivate the development of TurkishMedLLM as an integrated framework that combines Turkish medical data curation, semantic evidence retrieval, parameter-efficient model adaptation, source-grounded response generation, and multi-layer evaluation.

Based on these challenges, the study investigates four closely related research areas. First, it examines how heterogeneous Turkish medical resources can be transformed into traceable and reusable datasets suitable for both supervised model adaptation and retrieval-based evidence grounding. Second, it evaluates whether multilingual semantic retrieval can provide clinically relevant medical evidence for Turkish queries using embedding-based vector indexing. Third, it investigates how retrieval augmentation and QLoRA-based medical instruction fine-tuning affect the quality, faithfulness, and safety of Turkish medical response generation relative to the base Qwen3-8B model [11,12]. Fourth, it examines whether a multi-layer evaluation framework combining retrieval metrics, RAGAS [13], DeepEval [14], and clinical safety-oriented assessment [15] can characterize different aspects of medical LLM behavior. These research questions are addressed through the construction of a multi-source Turkish medical corpus, the development of a BGE-M3-based retrieval pipeline [16], QLoRA-based adaptation of Qwen3-8B, integration of retrieval-grounded response generation and safety-aware controls, and evaluation using complementary automated and expert-centred measures.

The resulting framework, TurkishMedLLM, is designed as a source-grounded and safety-aware Turkish medical LLM for clinician-supervised diagnostic decision support and digital health applications. The research pipeline is organized into independently auditable stages, including data ingestion, schema detection, quality filtering, text transformation, embedding generation, vector-store construction, retrieval evaluation, response generation, supervised fine-tuning, and safety-aware evaluation. This modular organization enables the contribution of individual components to be examined systematically while preserving source-level information for traceability and auditing. The curated resources comprise 210,791 Turkish medical question–answer pairs for supervised fine-tuning and 22,135 medical articles for retrieval. The article corpus is transformed into 206,166 retrieval-ready text chunks using the defined text-processing procedure, after which BGE-M3 embeddings are generated and indexed in ChromaDB [16,17] for semantic retrieval. The generative component is based on Qwen3-8B and is adapted using QLoRA, enabling systematic comparison among the base model, base model with retrieval augmentation, fine-tuned model, fine-tuned model with retrieval augmentation, and safety-aware fine-tuned retrieval-augmented configurations. The framework is therefore intended to provide a transparent and reproducible basis for investigating source-grounded Turkish medical language generation and clinician-supervised digital health support.

The main contributions of this study can be summarized as follows:

- A curated and traceable multi-source Turkish medical corpus comprising 210,791 question–answer pairs and 22,135 medical articles, prepared for complementary supervised fine-tuning and retrieval-based applications.
- A source-grounded retrieval pipeline that transforms medical articles into 206,166 retrieval-ready chunks and employs BGE-M3 embeddings with ChromaDB for semantic medical evidence retrieval.
- A Turkish medical LLM framework based on Qwen3-8B and QLoRA that integrates supervised medical instruction adaptation with retrieval-augmented response generation.
- A safety-aware response generation strategy designed to encourage evidence-supported responses, appropriate uncertainty communication, avoidance of unsupported diagnostic or medication claims, and escalation to qualified healthcare professionals when clinically appropriate.
- A multi-layer evaluation framework combining retrieval performance, response-quality assessment, automated RAG evaluation, clinical safety-oriented assessment, and clinician-centred evaluation to characterize the behavior of the proposed framework from complementary perspectives.

The organization of the paper is as follows. Section 2 addresses the related literature on medical LLMs, medical natural language processing (NLP), retrieval-augmented generation, vector-based retrieval, fine-tuning methods, and assessment of medical conversational AI systems. Section 3 presents the research methodology, including data construction, retrieval, response generation, safety-aware controls, fine-tuning, and evaluation design. Section 4 describes the implementation details of the proposed pipeline and its integration within the AIDCare digital health platform. Section 5 presents the experimental results, including dataset construction and quality analysis, retrieval performance, response generation, multi-layer evaluation, fine-tuning, ablation analysis, clinical safety assessment, and deployment efficiency. Section 6 provides a discussion of the main findings, their interpretation, the implications of the results, and the limitations and future research directions of the proposed framework. Section 7 summarizes the principal findings of the study, clarifies their significance within the evaluated experimental setting, and outlines directions for future research.

## 2. Related Work

### 2.1. Medical Large Language Models

Medical LLMs have demonstrated promising capabilities for clinical and biomedical question answering, symptom interpretation, and clinician-assisted diagnostic decision support [18,19]. One of the earliest influential studies evaluating medical LLMs on the MultiMedQA benchmark [18], which includes medical licensing examination questions, biomedical question answering, and consumer health queries, introduced Med-PaLM. The study demonstrated that instruction-tuned LLMs can achieve competitive performance across diverse medical benchmarks and highlighted the importance of human evaluation for assessing factuality, comprehension, reasoning, potential harm, and bias. Although Med-PaLM showed considerable promise for supporting clinical information retrieval and diagnostic reasoning, its responses were not consistently equivalent to those of healthcare professionals, particularly with respect to clinical safety, reliability, and the management of diagnostically complex cases. Med-PaLM 2 further improved medical question answering through stronger foundation models, medical-domain adaptation, and optimized prompting strategies. It achieved competitive performance across multiple medical benchmarks while generating higher-quality long-form consumer health responses. Nevertheless, challenges related to transparency, source traceability, localized clinical deployment, and support for low-resource medical languages remained largely unresolved.

Domain-specific pretraining has also demonstrated significant benefits for biomedical language models such as BioGPT and PubMedBERT [20]. BioGPT was pretrained on large-scale biomedical literature and substantially improved biomedical text mining and question answering, whereas PubMedBERT was trained from scratch using PubMed abstracts and full-text articles, leading to strong performance across a range of biomedical language understanding tasks [21,22]. However, BioGPT primarily targets biomedical literature, whereas PubMedBERT is an encoder-only model designed for language understanding rather than generative diagnostic assistance. Consequently, neither model directly addresses Turkish patient-oriented medical question answering, source-grounded retrieval-augmented generation (RAG), or clinically cautious diagnostic decision support. Existing research consistently demonstrates that domain adaptation is essential for trustworthy medical AI systems [23]. However, most state-of-the-art medical LLMs remain English-centric, are primarily evaluated using standardized benchmark datasets, and provide limited support for source-grounded, transparent, and clinically deployable diagnostic workflows in low-resource languages. Motivated by these limitations, our proposed TurkishMedLLM specializes in Turkish medical knowledge while integrating source traceability, retrieval grounding, multilingual vector embeddings, QLoRA-based parameter-efficient fine-tuning, and multi-layer safety evaluation to support clinician-supervised diagnostic decision support.

### 2.2. Medical NLP and Low-Resource Medical Language Processing

Medical natural language processing (NLP) for low-resource languages remains considerably less developed than English biomedical NLP, particularly for clinical question answering, symptom interpretation, retrieval-augmented diagnostic decision support, and trustworthy medical response generation [24]. TurkMedNLI represents an important step toward Turkish medical NLP by introducing a medical natural language inference dataset constructed through an LLM-assisted translation pipeline [25]. The study successfully preserved medical terminology, abbreviations, and semantic consistency across languages. However, its primary focus was natural language inference rather than Turkish medical question answering, source-grounded response generation, retrieval-augmented diagnostic support, or clinician-oriented medical LLM applications.

The TUSGPT-TR Medical Dataset [26] and the Zenodo Turkish Medical Question–Answer Dataset [27] provide valuable resources for supervised fine-tuning of Turkish medical LLMs. These datasets facilitate the adaptation of instruction-following models for Turkish patient communication, clinical question answering, and diagnostic decision-support scenarios. Nevertheless, raw datasets cannot be directly used for reliable medical AI because schema inconsistencies, duplicate records, missing fields, noisy text, short responses, and limited source traceability may adversely affect response quality, factual reliability, and clinical safety.

The TUSGPT-TR-Medical-Dataset-v1 [26], comprising 55,465 high-quality Turkish medical question–answer pairs, provides a specialized resource for supervised fine-tuning of Turkish medical language models. The dataset covers a broad spectrum of medical disciplines, including basic sciences such as pharmacology, pathology, anatomy, and physiology, as well as clinical specialties including internal medicine, surgery, pediatrics, obstetrics and gynecology, emergency medicine, neurology, oncology, and radiology. Such diversity makes the dataset valuable for developing domain-specific medical language models capable of supporting evidence-based clinical communication and diagnostic decision support. As with other instruction-tuning datasets, careful assessment of data quality, diversity, and source provenance remains essential to ensure the reliability and generalizability of downstream medical AI systems.

The Türkçe Tıbbi Soru–Cevap Veri Seti (Turkish Medical Question–Answer Dataset) [27], comprising 167,732 physician-answered health-related question–answer pairs, provides a large-scale Turkish medical resource for supervised fine-tuning and healthcare-oriented NLP applications. The dataset spans multiple categories, including general health, diseases, symptoms, diagnostics, treatment methods, and medications. Consequently, it offers broad coverage of patient-oriented medical information relevant to clinical communication and diagnostic support. However, similar to other large-scale web-sourced datasets, comprehensive pre-processing remains necessary to address noise, inconsistencies, and limited source traceability that may otherwise compromise model reliability and clinical trustworthiness.

The Turkish Hospital Medical Articles dataset [28], comprising 24,612 Turkish-language medical articles collected from 14 major hospitals and healthcare providers in Türkiye, provides a comprehensive corpus for medical NLP research and retrieval-augmented medical LLMs. The dataset contains full-length articles covering disease information, diagnostic procedures, medical treatments, preventive healthcare, wellness, and general medical guidance. Each record includes the article title, full text, publication date, update date, and original source URL, making the dataset suitable for applications such as semantic retrieval, clinical information retrieval, question answering, summarization, domain-specific pretraining, and retrieval-augmented diagnostic decision support. Since the corpus is compiled from publicly available hospital websites, appropriate pre-processing and quality assessment remain essential to ensure consistency, reduce redundancy, and preserve source traceability.

Before these documents can be utilized within a retrieval-augmented generation (RAG) framework, they must be segmented into retrieval-ready chunks, transformed into semantic vector embeddings, and indexed for efficient retrieval. TurkishMedLLM addresses this gap by constructing two complementary medical corpora: a cleaned instruction-tuning dataset for supervised fine-tuning and a retrieval-ready hospital article corpus for source-grounded retrieval, clinically relevant evidence retrieval, and trustworthy diagnostic decision support.

### 2.3. Retrieval-Augmented Generation for Medical Question Answering

Retrieval-augmented generation (RAG) has emerged as a promising approach for improving the factual accuracy, transparency, and trustworthiness of medical LLM responses by grounding generated content in external medical knowledge sources [29]. In a RAG framework, clinically relevant documents are retrieved from an external knowledge base and incorporated into the model’s context before response generation. This approach is particularly valuable in medical applications because the knowledge encoded within LLM parameters may become outdated, incomplete, or insufficient for supporting evidence-based clinical decision making. By incorporating retrieved medical evidence during inference, RAG reduces the likelihood of hallucinations, improves factual consistency, and enhances the transparency and verifiability of generated responses [30,31]. Consequently, RAG has been increasingly adopted for medical question answering, symptom interpretation, disease-related information retrieval, patient education, chronic disease self-management, medication guidance, and clinician-assisted diagnostic decision support [32,33].

Despite these advantages, retrieval augmentation alone does not guarantee clinically reliable or diagnostically appropriate responses [32]. The quality of generated responses depends on multiple factors, including the quality of the retrieval corpus, document chunking strategy, embedding model, vector database, retrieval configuration, prompt design, and safety mechanisms. Retrieved passages may be incomplete, irrelevant, or insufficiently aligned with the user’s clinical context, while the language model may fail to appropriately incorporate the retrieved evidence into its diagnostic reasoning. These challenges become even more pronounced for Turkish because of its rich morphology, flexible word formation, and the coexistence of formal medical terminology with colloquial patient language. Developing an effective Turkish medical RAG system therefore requires high-quality multilingual semantic embeddings, carefully curated medical knowledge sources, robust retrieval strategies, and clinically relevant evidence retrieval. To address these challenges, TurkishMedLLM constructs a source-grounded Turkish medical retrieval corpus using hospital medical articles and physician-validated question–answer pairs, generates multilingual semantic representations using the BGE-M3 embedding model, indexes the corpus in ChromaDB, and performs retrieval validation prior to response generation. This design supports transparent, evidence-based, and clinician-supervised diagnostic decision support while preserving source traceability throughout the retrieval process.

### 2.4. Medical Retrieval Using Embedding Models and Vector Databases

Embedding models play a fundamental role in semantic medical retrieval by transforming clinical queries and medical documents into dense vector representations that preserve semantic similarity. High-quality embeddings are particularly important for retrieval-augmented medical LLMs because the relevance of retrieved evidence directly influences the quality, factual consistency, and clinical reliability of generated responses. BGE-M3 [34] is well suited for our Turkish medical LLM because it supports multilingual, multi-function, and multi-granularity retrieval [35]. Developed for dense, sparse, and multi-vector retrieval across more than 100 languages, including Turkish, BGE-M3 is capable of capturing semantic relationships between clinician-oriented and patient-oriented medical queries, thereby facilitating clinically relevant evidence retrieval for diagnostic decision support.

Although multilingual embedding models substantially improve semantic retrieval, embedding quality alone is insufficient to guarantee trustworthy medical information retrieval. Reliable diagnostic decision support also depends on high-quality source datasets, appropriate document pre-processing, meaningful document chunking, comprehensive metadata, and complete source traceability. Vector databases such as ChromaDB and FAISS enable efficient similarity search over large medical document collections; however, they still require duplicate removal, metadata preservation, retrieval validation, and downstream evaluation of clinical response quality. TurkishMedLLM employs ChromaDB as a persistent vector database and preserves chunk identifiers, source names, article titles, URLs, and parent-document metadata to maintain source-level traceability and support transparent, evidence-based retrieval for clinician-supervised diagnostic decision support.

### 2.5. Fine-Tuning of Open-Source LLMs with Parameter-Efficient Learning

Fine-tuning is a widely adopted approach for adapting general-purpose LLMs to domain-specific tasks by improving their ability to understand specialized terminology, follow domain-specific instructions, and generate contextually appropriate responses. In the medical domain, fine-tuning enhances the model’s capability to interpret clinical terminology, respond consistently to patient-oriented queries, and support evidence-based clinical communication and diagnostic decision support. However, conventional fine-tuning is computationally expensive and may introduce overfitting or catastrophic forgetting, particularly when heterogeneous medical datasets are used for adaptation [36].

To address these limitations, Quantized Low-Rank Adaptation (QLoRA) provides a parameter-efficient approach for fine-tuning quantized LLMs using a relatively small number of trainable parameters. QLoRA propagates gradients through a frozen 4-bit quantized model while updating only low-rank adapter weights, thereby substantially reducing memory requirements without sacrificing adaptation performance [37]. This makes QLoRA particularly suitable for TurkishMedLLM, enabling efficient adaptation of Qwen3-8B using a carefully curated Turkish medical question–answer dataset without modifying the full set of model parameters. Qwen3-8B was selected as the base model based on its multilingual capabilities, strong reasoning performance, and suitability for parameter-efficient adaptation, which are particularly relevant for understanding Turkish medical terminology, patient-centered language, and complex clinical queries. These characteristics made Qwen3-8B an appropriate foundation for the proposed Turkish medical framework, where Turkish language adaptation through QLoRA is combined with retrieval-based source grounding and safety-aware response generation [38]. To evaluate the contribution of parameter-efficient fine-tuning, TurkishMedLLM systematically compares five configurations: the base LLM, base retrieval-augmented generation (RAG), fine-tuned LLM, fine-tuned RAG, and safety-aware fine-tuned RAG. This comparative evaluation enables assessment of the impact of fine-tuning and retrieval grounding on the generation of clinically relevant, source-grounded, and diagnostically supportive responses.

### 2.6. Assessing Medical LLM Systems with Retrieval-Augmented Generation

One of the greatest challenges in retrieval-augmented medical LLMs is the reliable evaluation of generated responses, since a fluent and relevant answer may still be factually unsupported, clinically inappropriate, or potentially unsafe for diagnostic decision support [39]. Consequently, evaluating medical LLMs requires a comprehensive assessment framework that extends beyond conventional natural language generation metrics to include retrieval effectiveness, contextual relevance, factual faithfulness, source groundedness, clinical caution, diagnostic relevance, and patient safety.

RAGAS provides quantitative metrics including faithfulness, answer relevancy, context precision, and context recall to evaluate whether retrieved medical evidence is relevant and whether generated responses are adequately supported by the retrieved context [13]. DeepEval complements this evaluation by measuring answer relevancy and contextual relevancy, thereby helping distinguish whether performance limitations originate from the retrieval component or the language generation component [14]. In addition, OpenAI Evals and custom grader-based evaluation enable task-specific assessment of clinically important characteristics such as source groundedness, clinical caution, avoidance of unsupported diagnostic claims, appropriate emergency escalation, medication dosage safety, and Turkish language fluency [40].

Building upon these complementary evaluation methodologies, TurkishMedLLM adopts a multi-layer evaluation framework that systematically assesses both retrieval quality and response generation. The retrieval component is evaluated using hit-rate metrics and keyword-based relevance estimation, while the generation component is assessed using RAGAS and DeepEval. Finally, clinical safety-oriented graders evaluate generated responses with respect to factual support, source traceability, diagnostic appropriateness, clinical caution, and patient safety. This comprehensive evaluation framework provides a rigorous assessment of whether TurkishMedLLM can generate trustworthy, evidence-based responses that support clinician-supervised diagnostic decision support in digital healthcare applications. Table 1 compares representative medical LLM, Turkish medical NLP, retrieval, fine-tuning, and evaluation studies, highlighting the remaining gaps in Turkish source-grounded medical response generation. The identified gaps and the corresponding components introduced in TurkishMedLLM are further summarized in Table 2.

### 2.7. Research Gaps

The reviewed literature demonstrates that medical LLMs have achieved promising results in medical question answering, biomedical language understanding, retrieval-augmented generation (RAG), multilingual semantic retrieval, and parameter-efficient fine-tuning. However, several important research gaps remain before these technologies can reliably support clinician-supervised diagnostic decision support and digital healthcare applications.

First, the majority of existing medical LLMs are designed primarily for English-language healthcare environments, with comparatively limited attention given to low-resource medical languages such as Turkish. Consequently, localized patient communication, symptom interpretation, and evidence-based diagnostic support remain insufficiently explored. Second, existing studies typically investigate individual components of the medical LLM pipeline, such as retrieval, fine-tuning, or response generation, rather than providing a unified and fully traceable framework that integrates source-aware data ingestion, schema standardization, duplicate removal, quality filtering, retrieval corpus construction, supervised fine-tuning, retrieval augmentation, and comprehensive safety evaluation. Third, many existing medical RAG systems provide limited insight into how retrieval quality, contextual faithfulness, source grounding, parameter-efficient fine-tuning, and clinical safety collectively influence the reliability of generated responses. Furthermore, relatively few studies evaluate whether generated responses are appropriate for clinician-assisted diagnostic decision support through systematic multi-layer evaluation.

To address these limitations, TurkishMedLLM introduces a reproducible, source-grounded, retrieval-augmented, and safety-aware framework specifically designed for Turkish medical language processing. Rather than focusing solely on benchmark performance, retrieval effectiveness, or model fine-tuning in isolation, TurkishMedLLM integrates data curation, semantic retrieval, multilingual vector embeddings, QLoRA-based parameter-efficient fine-tuning, retrieval-augmented response generation, and comprehensive multi-layer evaluation within a single auditable pipeline. Accordingly, the contribution of TurkishMedLLM lies in the systematic integration and evaluation of these complementary components within a localized Turkish medical framework, rather than in proposing each underlying technique as a new algorithmic method. By emphasizing source traceability, factual faithfulness, clinical caution, and transparent evidence retrieval, the proposed framework supports trustworthy clinician-supervised diagnostic decision support and provides a practical foundation for future AI-assisted clinical diagnostic systems and digital healthcare applications in low-resource medical languages.

The novelty of TurkishMedLLM does not arise from introducing a new large language model, retrieval algorithm, or fine-tuning method in isolation, but from the systematic integration of these established techniques into a unified, source-grounded, and safety-aware framework specifically designed for Turkish medical language processing. As summarized in Table 1, existing studies primarily address individual aspects of medical LLM development, such as medical language modeling, Turkish medical NLP, multilingual retrieval, parameter-efficient fine-tuning, or RAG evaluation, while their integration into an auditable Turkish medical diagnostic-support workflow remains limited. Table 2 further summarizes how TurkishMedLLM addresses these gaps by combining source-traceable dataset construction, multilingual semantic retrieval using BGE-M3, QLoRA-based adaptation of Qwen3-8B, retrieval-augmented response generation, and multi-layer clinical safety evaluation within a single pipeline. Therefore, the contribution of this work lies in the integration, localization, traceability, and systematic evaluation of these components for Turkish clinician-supervised medical question answering and diagnostic decision support.

## 3. Research Methodology

The proposed research methodology follows a modular and reproducible framework in which medical data preparation, semantic retrieval, response generation, parameter-efficient fine-tuning, clinical safety control, and multi-layer evaluation are organized as interconnected yet independently auditable components. The overall objective of the framework is to develop a source-grounded Turkish medical large language model that supports clinician-supervised diagnostic decision support, symptom interpretation, and evidence-based medical question answering while maintaining transparency, traceability, and clinical caution. Figure 1 illustrates the complete methodological workflow of the proposed TurkishMedLLM architecture.

The pipeline begins with heterogeneous but authentic Turkish medical data sources and proceeds through data ingestion, schema standardization, quality filtering, dataset construction, supervised fine-tuning preparation, retrieval corpus generation, semantic embedding, vector indexing, retrieval-augmented response generation, safety-aware response control, and comprehensive multi-layer evaluation. To support both medical knowledge adaptation and evidence-grounded response generation, the framework employs two complementary processing streams: (i) a physician-oriented question–answer corpus for supervised fine-tuning and (ii) a hospital medical article corpus for retrieval-augmented generation (RAG). This dual-stream design enables the proposed framework to combine parametric medical knowledge with dynamically retrieved clinical evidence, thereby improving response faithfulness, source traceability, and support for clinician-supervised diagnostic decision making.

### 3.1. Problem Formulation

Let the collection of heterogeneous Turkish medical data sources be represented as

(1) $$X=\left\{X_{qa}^{\left(1\right)},X_{qa}^{\left(2\right)},X_{art}^{\left(3\right)}\right\}$$

where $X_{qa}^{\left(1\right)}$ and $X_{qa}^{\left(2\right)}$ denote two Turkish medical question–answer datasets, while $X_{art}^{\left(3\right)}$ denotes the Turkish hospital medical article dataset. Following data ingestion, schema standardization, duplicate removal, quality filtering, and pre-processing, the raw datasets are transformed into two structured corpora,

(2) $$C=\left\{C_{sft},C_{rag}\right\}$$

where $C_{sft}$ represents the supervised fine-tuning corpus used for adapting the language model, and $C_{rag}$ represents the retrieval corpus used for retrieval-augmented generation (RAG).

Given a user query *q*, the retrieval module retrieves the top-*k* most semantically relevant medical context chunks from the retrieval corpus,

(3) $$C_{q}=R\left(q,C_{rag},k\right)$$

where R(·) denotes the retrieval function, *k* represents the number of retrieved context chunks, and

(4) $$C_{q}=\left\{c_{1},c_{2},\ldots ,c_{k}\right\}$$

with each $c_{i}$ representing an individual retrieved medical document chunk that serves as supporting evidence for response generation.

The retrieved medical evidence is then supplied to the language model to generate a response,

(5) $$y=G_{\theta }\left(q,C_{q}\right)$$

where $G_{\theta }$ denotes the parameterized large language model, θ represents the trainable model parameters, and *y* denotes the generated Turkish medical response.

The objective of TurkishMedLLM is to generate responses that are relevant to the user’s medical query, faithful to the retrieved medical evidence, clinically cautious, and associated with minimal clinical risk. This objective is formulated as

(6) $$\begin{array}{cc} \max_{y}\;S\left(y,q,C_{q}\right)= & \lambda_{1}\mathrm{Rel}\left(y,q\right)+\lambda_{2}\mathrm{Faith}\left(y,C_{q}\right)\\ \; & +\lambda_{3}\mathrm{Safe}\left(y\right)-\lambda_{4}\mathrm{Risk}\left(y\right) \end{array}$$

where S(·) denotes the overall response quality score, Rel(y,q) measures the semantic relevance of the generated response to the user’s query, $\mathrm{Faith}(y,C_{q})$ measures the factual consistency of the generated response with the retrieved medical evidence, Safe(y) evaluates the degree of clinically cautious, evidence-based, and responsible response behavior, and Risk(y) quantifies undesirable behaviors such as unsupported diagnostic claims, unsafe medication recommendations, omission of emergency escalation advice, or hallucinated medical information. The coefficients $\lambda_{1}$, $\lambda_{2}$, $\lambda_{3}$, and $\lambda_{4}$ are non-negative weighting parameters that control the relative contribution of each objective component.

The optimization objective is intended to support clinician-supervised diagnostic decision support by encouraging evidence-grounded, clinically cautious, and transparent medical responses rather than autonomous diagnosis. This formulation emphasizes that TurkishMedLLM is designed to assist healthcare professionals and patients through trustworthy medical information retrieval and evidence-based response generation, while preserving clinical oversight and minimizing the risk of unsupported or potentially unsafe recommendations.

### 3.2. Data Source Design

The proposed TurkishMedLLM framework utilizes three publicly available Turkish medical datasets that are organized into two complementary data streams, as summarized in Table 3. The first stream consists of Turkish medical question–answer datasets that are used for supervised fine-tuning to enable the language model to learn clinically relevant instruction-following behavior, medical terminology, and patient-oriented communication patterns. The second stream consists of Turkish hospital medical articles that serve as the external knowledge base for retrieval-augmented generation (RAG), providing evidence-based medical information and source-grounded clinical context during inference. This dual-stream design enables TurkishMedLLM to combine parametric medical knowledge acquired through supervised fine-tuning with dynamically retrieved medical evidence, thereby improving factual consistency, source traceability, and clinician-supervised diagnostic decision support.

The selected datasets collectively cover a broad spectrum of medical knowledge, including diseases, symptoms, diagnostic procedures, treatment options, medications, preventive healthcare, and patient education. Since all datasets originate from publicly available Turkish medical resources, they provide an appropriate foundation for developing a localized medical LLM while preserving source-level traceability throughout the retrieval pipeline. Their complementary characteristics support both supervised learning and evidence-grounded response generation, making them suitable for trustworthy medical question answering and diagnostic decision-support applications.

The datasets used in this research were obtained from publicly available sources that are cited in Table 3. The TUSGPT-TR Medical Dataset and the Turkish Medical Question–Answer Dataset are publicly released research datasets, while the Turkish Hospital Medical Articles dataset was compiled by [28] from publicly accessible hospital websites in Türkiye. All datasets contain anonymized textual information and do not include personally identifiable patient information. The datasets were used in accordance with their respective licenses and intended research purposes.

### 3.3. Data Ingestion and Schema Standardization

The data ingestion stage transforms heterogeneous Turkish medical datasets stored in JSON, CSV, and Parquet formats into a unified document representation suitable for supervised fine-tuning and retrieval-augmented generation. Since the datasets originate from multiple sources with different schemas, field names, and storage formats, a standardized representation is required to ensure data consistency, source traceability, and reproducible downstream processing. For each raw record $x_{i}$, the transformation function T(·) converts the original record into a standardized document,

(7) $$d_{i}=T\left(x_{i},M_{i}\right)$$

where T(·) denotes the schema transformation function, $d_{i}$ represents the standardized document, and $M_{i}$ denotes the associated metadata, including the source identifier, dataset name, file name, row index, dataset type, data split, and intended downstream application. Preserving this metadata enables complete source-level traceability throughout the retrieval, fine-tuning, and evaluation pipeline.

For medical question–answer datasets, each standardized document is represented as

(8) $$d_{i}^{qa}=\left\{q_{i},a_{i},M_{i}\right\}$$

where $q_{i}$ denotes the Turkish medical question, $a_{i}$ denotes the corresponding medically relevant answer, and $M_{i}$ stores the associated metadata.

For hospital medical articles, each standardized document is represented as

(9) $$d_{i}^{art}=\left\{t_{i},M_{i}\right\}$$

where $t_{i}$ denotes the medical article text and $M_{i}$ contains the associated metadata required for source traceability and retrieval.

Because the source datasets follow different schema conventions, flexible schema detection is performed to automatically identify semantically equivalent fields. Question fields are detected using keywords such as *question*, *soru*, *input*, *prompt*, *instruction*, and *hasta_sorusu*. Similarly, answer fields are identified using keywords including *answer*, *cevap*, *output*, *response*, and *completion*. When direct field matching is unsuccessful, a length-based heuristic is applied, where the shorter textual field is assigned as the question and the longer textual field is assigned as the answer. All schema detection decisions are automatically recorded in processing reports to facilitate auditing, reproducibility, and quality assurance throughout the data preparation pipeline.

### 3.4. Dataset Selection, Duplicate Removal, and Quality Filtering

To ensure the construction of a reliable and reproducible medical corpus, TurkishMedLLM employs a systematic data selection and quality control strategy before fine-tuning and retrieval dataset construction. The objective of this stage is to eliminate redundant records, standardize dataset versions, and remove low-quality samples that may negatively affect retrieval accuracy, response generation, and clinician-supervised diagnostic decision support.

A dataset selection policy is first applied to avoid duplicate processing of equivalent dataset releases. When both complete and pre-defined training, validation, or testing splits are available, the split datasets are selected to preserve the intended experimental partitioning. Likewise, when both CSV and Parquet versions of the same dataset exist, the Parquet version is preferred because of its efficient storage format and faster large-scale data processing.

Duplicate detection is performed independently for the question–answer corpus and the hospital article corpus. For question–answer records, the normalized duplicate key is defined as

(10) $$K_{qa}\left(i\right)=\mathrm{norm}\left(q_{i}\right)\;\left|\right|\;\mathrm{norm}\left(a_{i}\right)$$

where norm(·) denotes the text normalization function and || represents string concatenation. Duplicate removal follows the rule

(11) $$d_{i}^{qa}=\left\{\begin{array}{cc} \mathrm{retain}, & K_{qa}\left(i\right)\notin K_{qa}\\ \mathrm{discard}, & K_{qa}\left(i\right)\in K_{qa} \end{array}\right.$$

where $K_{qa}$ denotes the set of previously observed normalized question–answer keys.

Similarly, duplicate detection for hospital medical articles is performed using

(12) $$K_{art}\left(i\right)=\mathrm{norm}\left(t_{i}\right)$$

where $t_{i}$ denotes the article text and norm(·) removes formatting variations before duplicate comparison.

After duplicate removal, quality filtering is applied to eliminate incomplete or weakly informative records. The filtering criteria require each medical question to contain at least 10 characters, each answer at least 30 characters, each combined question–answer pair at least 50 characters, and each hospital medical article at least 200 characters. These empirically selected thresholds remove extremely short, incomplete, or noisy records while preserving medically meaningful content for both supervised learning and retrieval.

Following dataset selection, duplicate removal, and quality filtering, the final processed corpus contains 232,926 unique medical documents, comprising 210,791 Turkish medical question–answer pairs and 22,135 hospital medical articles. Overall, 9139 low-quality or duplicate records were removed during pre-processing. This quality control process improves dataset consistency, reduces redundant training samples, enhances retrieval efficiency, and contributes to more reliable, source-grounded, and clinically relevant response generation.

To minimize the possibility of information leakage across experimental stages, a partition-aware data control procedure was applied throughout dataset preparation. The supervised fine-tuning data, validation data where applicable, retrieval corpus, and evaluation queries were maintained as distinct experimental inputs, with source identifiers, dataset names, row indices, split assignments, and intended downstream use preserved in the associated metadata. The question–answer records used for supervised fine-tuning were not used as evaluation queries, while the hospital medical articles were maintained as the retrieval corpus. Evaluation queries were reserved for retrieval and response assessment and were not incorporated into model adaptation. Duplicate detection was performed before downstream dataset construction using normalized text representations to prevent identical records from being retained across separate experimental inputs. This procedure provided traceable separation between model-development data, retrieval knowledge, and held-out evaluation inputs.

### 3.5. Supervised Fine-Tuning Dataset Preparation

Following pre-processing and quality filtering, the cleaned Turkish medical question–answer corpus is transformed into an instruction-tuning dataset suitable for supervised fine-tuning of the base large language model. Instruction tuning enables the model to learn medically appropriate response patterns, domain-specific terminology, and patient-oriented communication while preserving the original source information for traceability. Each training sample is represented as

(13) $$s_{i}=\left\{{\mathrm{inst}}_{i},{\mathrm{input}}_{i},{\mathrm{output}}_{i},M_{i}\right\}$$

where ${\mathrm{inst}}_{i}$ denotes the instruction prompt that guides the model’s expected behavior, ${\mathrm{input}}_{i}$ represents the Turkish medical question, ${\mathrm{output}}_{i}$ denotes the corresponding medically relevant reference answer, and $M_{i}$ contains the associated metadata, including source information required for dataset traceability and auditing.

The complete supervised fine-tuning corpus is defined as

(14) $$C_{sft}=\left\{s_{1},s_{2},\ldots ,s_{N}\right\}$$

where N=210,791 denotes the total number of instruction-tuning samples after pre-processing and quality filtering.

The resulting supervised fine-tuning corpus provides high-quality Turkish medical instruction–response examples that enable the language model to adapt to localized medical terminology, patient-oriented communication, and clinically relevant question answering. Although the model learns domain-specific medical knowledge during fine-tuning, the generated responses are subsequently combined with retrieval-augmented generation to incorporate current source-grounded medical evidence, thereby improving factual consistency, transparency, and support for clinician-supervised diagnostic decision support.

### 3.6. Text Transformation and Retrieval Dataset Construction

To support retrieval-augmented generation (RAG), the cleaned hospital medical articles are transformed into retrieval-ready text chunks while preserving semantic coherence and source traceability. Since complete medical articles often exceed the context window of large language models, each article is recursively divided into smaller overlapping segments that can be efficiently indexed and retrieved during inference. For each medical article $t_{i}$, the splitting function is defined as

(15) $$\mathrm{Split}\left(t_{i}\right)=\left\{c_{i,1},c_{i,2},\ldots ,c_{i,l_{i}}\right\}$$

where Split(·) denotes the recursive text splitting function, $c_{i,j}$ represents the *j*-th retrieval chunk generated from article *i*, and $l_{i}$ denotes the total number of chunks produced from article $t_{i}$.

The complete retrieval corpus is then constructed as

(16) $$C_{rag}=\overset{A}{\underset{i=1}{⋃}}\mathrm{Split}\left(t_{i}\right)$$

where A=22,135 denotes the total number of hospital medical articles included in the retrieval corpus. Recursive text splitting is performed using a chunk size of 1000 characters with an overlap of 150 characters between adjacent chunks. The overlap preserves semantic continuity across neighboring text segments and reduces the likelihood of losing clinically important information located near chunk boundaries. This strategy improves semantic retrieval by ensuring that disease descriptions, diagnostic procedures, treatment recommendations, and other clinically relevant information remain contextually complete during retrieval. Following chunk generation, the retrieval corpus consists of 206,166 retrieval-ready medical text chunks, while 1776 low-quality text fragments are discarded during pre-processing. Throughout this process, metadata associated with every chunk—including the original article identifier, source, title, and document information—are preserved to maintain complete source traceability. This enables TurkishMedLLM to retrieve evidence-grounded medical context that supports transparent, clinically relevant, and clinician-supervised diagnostic decision support during response generation.

### 3.7. Embedding Generation and Vector Store Construction

Following retrieval corpus construction, each retrieval-ready medical text chunk is converted into a dense semantic vector representation to enable efficient similarity-based retrieval. Dense embeddings capture the semantic meaning of medical concepts beyond exact keyword matching, allowing clinically related documents to be retrieved even when different medical terminology or patient-oriented language is used. For each retrieval chunk $c_{i}$, the embedding function is defined as

(17) $$v_{i}=E\left(c_{i}\right)$$

where E(·) denotes the embedding function implemented using the multilingual BGE-M3 embedding model through Ollama, $v_{i}\in R^{d}$ represents the dense embedding vector corresponding to chunk $c_{i}$, and *d* denotes the embedding dimensionality produced by the model.

The complete vector index is constructed as

(18) $$V={\left\{(v_{i},c_{i},M_{i})\right\}}_{i=1}^{m}$$

where m=206,166 denotes the total number of retrieval-ready medical text chunks, $c_{i}$ represents the corresponding text chunk, and $M_{i}$ contains the associated metadata, including the original source, article title, document identifier, and other information required for source traceability.

ChromaDB is employed as the persistent vector database to store all 206,166 embedded medical text chunks, while BGE-M3 is used as the multilingual embedding model through Ollama to support local embedding generation. Embeddings are generated in batches of 500 chunks to improve computational efficiency and memory utilization during large-scale indexing. In addition, checkpointing and LangSmith tracing are enabled throughout the indexing process to facilitate fault recovery, monitoring, reproducibility, and auditability.

The resulting vector database enables efficient semantic retrieval of clinically relevant medical evidence during retrieval-augmented generation. By combining multilingual semantic embeddings with complete source metadata, TurkishMedLLM retrieves evidence that is both semantically relevant and fully traceable, thereby supporting transparent, evidence-grounded, and clinician-supervised diagnostic decision support.

### 3.8. Retriever Construction and RAG Generation

During inference, each user medical query is converted into a semantic embedding and matched against the indexed retrieval corpus to identify the most relevant supporting medical evidence. This retrieval step enables the language model to generate responses that are grounded in external medical knowledge rather than relying solely on its parametric memory.

Given a user query *q*, the corresponding query embedding is computed as

(19) $$v_{q}=E\left(q\right)$$

where E(·) denotes the multilingual BGE-M3 embedding function introduced in Section 3.7, and $v_{q}\in R^{d}$ represents the dense semantic embedding of the input medical query.

The semantic similarity between the query embedding and each indexed document embedding is computed using cosine similarity,

(20) $$\mathrm{sim}\left(v_{q},v_{i}\right)=\frac{v_{q}^{⊤}v_{i}}{∥v_{q}∥\;∥v_{i}∥}$$

where $v_{i}$ denotes the embedding vector of the *i*-th retrieval chunk and ∥·∥ represents the Euclidean norm.

The retrieval module then selects the top-*k* highest-ranked medical text chunks according to their similarity scores,

(21) $$C_{q}=\mathrm{TopK}\left({\left\{\mathrm{sim}(v_{q},v_{i})\right\}}_{i=1}^{m},k\right)$$

where *k* denotes the number of retrieved context chunks and

(22) $$C_{q}=\left\{c_{1},c_{2},\ldots ,c_{k}\right\}$$

represents the ordered set of retrieved medical evidence supplied to the language model.

The retrieval-augmented generation module subsequently generates the final medical response according to

(23) $$y=G_{\theta }\left(q,C_{q},P\right)$$

where $G_{\theta }$ denotes the fine-tuned large language model, $C_{q}$ represents the retrieved medical evidence, *P* denotes the prompt template, and *y* is the generated Turkish medical response.

The prompt template instructs the language model to respond exclusively in Turkish, utilize only the retrieved medical evidence when formulating answers, avoid unsupported or hallucinated medical claims, refrain from providing definitive diagnoses or unsupported medication dosage recommendations, encourage consultation with qualified healthcare professionals when clinically appropriate, and explicitly acknowledge situations in which the available evidence is insufficient. These prompt constraints encourage transparent, evidence-grounded, and clinically cautious responses that support clinician-supervised diagnostic decision support rather than autonomous medical diagnosis.

When the retrieval layer does not provide sufficiently relevant evidence for a medical query, the prompt instructs the model not to infer or construct an unsupported medical recommendation from its parametric knowledge. Instead, the model is required to explicitly indicate that adequate evidence is unavailable and recommend consultation with a qualified healthcare professional when appropriate. This source-availability constraint is intended to reduce hallucinated medical information and prevent unsupported diagnostic or treatment recommendations when the retrieved evidence is insufficient.

### 3.9. Safety-Aware Response Control

Following response generation, TurkishMedLLM applies a safety-aware control layer to assess whether the generated response satisfies predefined clinical safety requirements before it is presented to the user. The objective of this stage is to reduce the risk of hallucinated medical information, unsupported diagnostic claims, unsafe medication recommendations, and omission of appropriate emergency escalation guidance. The safety evaluation considers the consistency of the generated response with the retrieved medical evidence, the appropriateness of clinical recommendations, and the overall level of patient safety.

The final response selection is defined as

(24) $$\hat{y}=\left\{\begin{array}{cc} y, & \mathrm{Safe}(y,C_{q})\geq \tau \\ y_{\mathrm{safe}}, & \mathrm{Safe}(y,C_{q})<\tau \end{array}\right.$$

where $\hat{y}$ denotes the final response delivered to the user, *y* is the response generated by the retrieval-augmented language model, $y_{\mathrm{safe}}$ represents a revised or cautionary response generated when the original response does not satisfy the required safety criteria, $\mathrm{Safe}(y,C_{q})$ denotes the clinical safety assessment function that evaluates the generated response using the retrieved medical evidence $C_{q}$, and τ represents the predefined safety threshold. The safety assessment verifies whether the generated response is adequately supported by the retrieved medical evidence, avoids unsupported diagnostic claims, refrains from providing unsupported medication dosage recommendations, includes appropriate uncertainty when evidence is insufficient, and recommends consultation with qualified healthcare professionals whenever clinically necessary. For high-risk clinical scenarios, including chest pain, stroke symptoms, severe breathing difficulty, severe hypoglycemia, pregnancy-related complications, and pediatric emergencies, the safety layer prioritizes immediate emergency escalation guidance instead of providing potentially unsafe or misleading medical advice. This safety-aware response control mechanism ensures that TurkishMedLLM functions as an evidence-grounded clinical decision-support system rather than an autonomous diagnostic system. By enforcing source-grounded responses, clinical caution, transparent uncertainty, and appropriate escalation recommendations, the framework promotes trustworthy and clinically responsible assistance for clinician-supervised diagnostic decision support and digital healthcare applications.

### 3.10. Supervised Fine-Tuning Strategy

The base model, Qwen3-8B, is adapted to the Turkish medical domain through supervised fine-tuning using Quantized Low-Rank Adaptation (QLoRA). Parameter-efficient fine-tuning enables the model to learn Turkish medical terminology, patient-oriented communication patterns, and clinically relevant response behavior without updating the complete set of pretrained model parameters. The QLoRA configuration adopted in this study is summarized in Table 4.

The supervised fine-tuning objective minimizes the negative log-likelihood of the reference answer tokens conditioned on the instruction prompt and the corresponding medical question,

(25) $$L_{sft}=-\sum_{t=1}^{T}\log p_{\theta }\left(y_{t}|y_{<t},{\mathrm{inst}}_{i},{\mathrm{input}}_{i}\right)$$

where $L_{sft}$ denotes the supervised fine-tuning loss, *T* is the total number of output tokens, $y_{t}$ represents the target token at decoding step *t*, $y_{<t}$ denotes all previously generated target tokens, ${\mathrm{inst}}_{i}$ is the instruction prompt, ${\mathrm{input}}_{i}$ is the corresponding Turkish medical question, $p_{\theta }\left(\cdot \right)$ represents the conditional probability predicted by the language model, and θ denotes the trainable model parameters.

QLoRA adapts the frozen quantized base model by learning low-rank weight updates instead of modifying the original model parameters. The adapted weight matrix is expressed as

(26) $$W^{\prime }=W+\Delta W=W+BA$$

where *W* denotes the frozen pretrained weight matrix, $W^{\prime }$ is the adapted weight matrix after fine-tuning, ΔW represents the learned parameter update, and *A* and *B* are trainable low-rank matrices whose product approximates the full weight update while substantially reducing the number of trainable parameters and memory requirements.

The supervised fine-tuning process uses 210,791 cleaned Turkish medical question–answer pairs to specialize the base model for localized medical question answering and clinically relevant communication. The complete experimental configuration, including the base model, QLoRA parameters, training strategy, and evaluation settings, is summarized in Table 4. The resulting fine-tuned model is subsequently integrated with the retrieval-augmented generation pipeline so that learned medical knowledge is complemented by dynamically retrieved, source-grounded medical evidence during inference. This combination improves factual consistency, response transparency, and support for clinician-supervised diagnostic decision support while avoiding reliance solely on the model’s parametric knowledge.

### 3.11. Evaluation and Ablation Design

The overall experimental design is illustrated in Figure 2. TurkishMedLLM is evaluated using five experimental configurations that progressively introduce retrieval augmentation, supervised fine-tuning, and safety-aware prompting. The complete ablation settings are summarized in Table 5. The evaluated configurations include the base Qwen3-8B model, base Qwen3-8B with retrieval-augmented generation (RAG), fine-tuned Qwen3-8B, fine-tuned Qwen3-8B with RAG, and fine-tuned Qwen3-8B with RAG and safety-aware prompting.

The effectiveness of the retrieval module is first evaluated using Hit@*k*. For a test query $q_{j}$, the retrieval success is defined as

(27) $$\mathrm{Hit}@k\left(q_{j}\right)=\left\{\begin{array}{cc} 1, & \exists \;c_{i}\in C_{q_{j}}^{\left(k\right)}:\mathrm{rel}\left(c_{i},q_{j}\right)>0\\ 0, & \mathrm{otherwise} \end{array}\right.$$

where $q_{j}$ denotes the *j*-th evaluation query, $C_{q_{j}}^{\left(k\right)}$ represents the set of the top-*k* retrieved medical text chunks for query $q_{j}$, and $\mathrm{rel}(c_{i},q_{j})$ is the relevance function indicating whether chunk $c_{i}$ contains clinically relevant information for answering the query.

The overall retrieval performance is computed as

(28) $$\mathrm{HitRate}@k=\frac{1}{Q}\sum_{j=1}^{Q}\mathrm{Hit}@k\left(q_{j}\right)$$

where *Q* denotes the total number of evaluation queries. A higher HitRate@*k* indicates that clinically relevant medical evidence is successfully retrieved before response generation.

Following retrieval evaluation, the generated medical responses are assessed using a multi-layer evaluation protocol summarized in Table 6. RAGAS evaluates faithfulness, answer relevancy, context precision, and context recall to determine whether the generated responses remain grounded in the retrieved medical evidence. DeepEval independently evaluates answer relevancy, faithfulness, contextual relevancy, contextual precision, and contextual recall to validate the robustness of the retrieval-augmented generation pipeline. Finally, clinical safety graders assess source-groundedness, medication safety, emergency escalation behavior, avoidance of unsupported diagnostic claims, Turkish fluency, and patient readability to determine the suitability of generated responses for clinician-supervised diagnostic decision support.

The combined evaluation protocol enables separate assessment of retrieval quality, response generation quality, evidence grounding, and clinical safety, thereby providing a comprehensive evaluation framework for TurkishMedLLM. The experimental configurations used for the ablation study are presented in Table 5, while the evaluation metrics employed at each stage are summarized in Table 6.

### 3.12. Traceability and Reproducibility

To ensure transparency, reproducibility, and auditability, complete traceability is maintained throughout every stage of the TurkishMedLLM pipeline. Each processed record preserves its associated metadata, including the source identifier, original file name, row index, dataset type, dataset split, and intended downstream use. Similarly, every retrieval-ready article chunk retains its parent document identifier, chunk identifier, article title, source URL, and additional metadata required to establish a direct link between generated responses and their original medical information sources.

Each module of the proposed framework produces structured processing reports that document the execution of the corresponding stage. These reports include selected and ignored input files, detected dataset schemas, pre-processing statistics, valid and discarded records, duplicate removal summaries, chunking parameters, embedding configurations, vector-store construction logs, retrieval queries, retrieved context chunks, generated responses, and evaluation results. The preservation of these intermediate artifacts enables systematic verification of every processing stage and facilitates debugging, performance analysis, and independent replication of the complete experimental pipeline.

By maintaining source-level provenance from the original datasets to the final generated responses, TurkishMedLLM provides transparent evidence tracking for retrieval-augmented generation and clinical response generation. This traceability framework supports reproducible experimentation, systematic auditing, and verification of evidence-grounded medical responses, thereby improving the reliability of clinician-supervised diagnostic decision support and future translational digital health applications.

## 4. Implementation

### 4.1. mHealth Workflow

The mHealth workflow integrates TurkishMedLLM into patient-facing mobile application and a clinician-facing web dashboard to support evidence-grounded medical question answering under human clinical supervision. The workflow is designed to connect patient queries with source-grounded medical evidence, safety-aware response generation, and clinician validation before the final response is returned to the patient. This human-in-the-loop design provides an additional layer of clinical oversight and helps ensure that AI-generated responses remain evidence-grounded, clinically cautious, and appropriate for patient-facing communication.

As illustrated in Figure 3, the workflow begins when a patient submits a medical query through the mHealth application. The query is processed and passed to TurkishMedLLM, where the query is embedded using BGE-M3 and matched against the ChromaDB vector database to retrieve relevant medical evidence. The retrieved evidence is subsequently combined with the patient query through a safety-aware prompting mechanism and provided to the fine-tuned Qwen3-8B model for response generation.

The generated response is then transmitted to the clinician web dashboard for human-in-the-loop (HITL) review. The clinician can assess the response for clinical appropriateness, factual reliability, completeness, and safety, and can correct or supplement the generated information when necessary. Following clinician validation, the approved or corrected response is returned to the patient through the mHealth application. This workflow ensures that TurkishMedLLM functions as a clinician-supervised decision-support and patient-education component rather than as an autonomous diagnostic system.

### 4.2. Integration in AIDCare Platform

To demonstrate the practical applicability of TurkishMedLLM in a real-world clinical setting, the proposed solution has been integrated into our AI-assisted Diabetes Care (AIDCare) mHelath application. The AIDCare solution contains a clinician-supervised digital health platform that supports patient care, symptom interpretation, and diagnostic decision support for diabetes management. Rather than replacing healthcare professionals, the system assists both patients and clinicians by providing evidence-grounded medical responses supported by retrieved medical knowledge. As illustrated in Figure 4, the patient interacts with the AI assistant through the AIDCare mobile application by submitting questions or describing their symptoms in Turkish. The mobile app communicates with the AI server through RESTful APIs. Upon receiving a request, the backend executes the TurkishMedLLM retrieval-augmented generation pipeline, which retrieves relevant medical evidence from the vector database using BGE-M3 embeddings before generating a response with the fine-tuned Qwen3-8B medical LLM. The generated response is subsequently returned to the mobile application as well as shared with the related clinician on his web-based dashboard.

The clinician dashboard enables a key feature of the implemented system, namely the Human-in-the-Loop (HITL) mechanism. As shown in Figure 5, each patient query and its corresponding AI-generated response are simultaneously presented on the clinician dashboard. Healthcare professionals can review the generated response, assess its clinical appropriateness, identify and correct inaccurate or incomplete information when necessary, and provide additional recommendations based on their professional judgment. This clinician oversight helps ensure that the information provided to patients is clinically relevant, sufficiently comprehensive, and appropriate for their individual needs, thereby providing an additional layer of safety and clinical validation to the AI-assisted decision-support process. This clinician verification process also strengthens patient safety, supports continuous quality improvement of the AI system, and ensures that TurkishMedLLM functions as a clinical decision-support system rather than an autonomous diagnostic tool.

### 4.3. Evaluation Implementation

The evaluation framework combines automated benchmarking with expert clinical assessment to provide a comprehensive evaluation of TurkishMedLLM. The automated evaluation pipeline measures retrieval effectiveness, response grounding, language quality, and clinical safety, whereas the expert evaluation assesses the practical clinical usefulness and reliability of the generated responses.

The retriever evaluation module uses a curated set of Turkish medical queries covering diabetes, hypertension, pediatric fever, cardiovascular symptoms, asthma, gestational diabetes, kidney disease, stroke, medication safety, hypoglycemia, endocrine disorders, neurological conditions, gastrointestinal disorders, respiratory diseases, and anemia. For each query, the five highest-ranked retrieval results are stored together with their associated metadata and manually categorized as relevant, partially relevant, or irrelevant according to predefined clinical relevance criteria.

The response generation module is evaluated using RAGAS, DeepEval, and custom clinical safety graders. Automated evaluation measures include faithfulness, answer relevancy, context precision, context recall, contextual relevancy, source-groundedness, medication safety, emergency escalation correctness, unsupported-claim avoidance, Turkish fluency, and patient readability.

For the clinical safety evaluation, the clinical caution score was calculated using a predefined set of safety-oriented criteria applied to the generated medical responses. Each response was assessed with respect to (i) avoidance of unsupported diagnostic claims, (ii) appropriate expression of uncertainty when the available evidence was insufficient, (iii) avoidance of unsupported medication dosage recommendations, (iv) recommendation of consultation with a qualified healthcare professional when clinically necessary, and (v) appropriate escalation guidance for high-risk symptoms. The clinical caution score was computed as the mean normalized score across the evaluated responses for these criteria, with higher values indicating more consistent adherence to the predefined clinical caution requirements. The resulting clinical caution score of 0.93 therefore represents the aggregated performance of the evaluated responses with respect to these safety-oriented criteria. The same evaluation procedure was applied consistently across the clinical safety dimensions reported in the multi-layer evaluation.

To further characterize the reliability of the reported evaluation results, performance measures were interpreted together with the variability observed across the evaluation procedure rather than as single-point estimates alone. For the fine-tuning experiments, performance was monitored across training epochs using both training and validation losses and ROUGE-based metrics, while the retrieval evaluation was conducted on a predefined set of 15 Turkish medical queries with multiple retrieval positions assessed for each query. The reported results are therefore interpreted in the context of the corresponding evaluation samples and observed variation. Where repeated measurements were available, mean values and standard deviations are reported to describe performance variability. Given the heterogeneous nature of the automated evaluation metrics and the absence of a common reference distribution across RAGAS, DeepEval, retrieval, and clinical safety measures, the reported scores are presented as descriptive evaluation results rather than as evidence of statistically significant clinical performance differences.

To provide an independent expert-based validation of the retrieval performance, an additional evaluation was conducted using a separate set of 100 Turkish medical questions covering multiple clinical specialties. The questions were submitted through the TurkishMedLLM web panel, and the corresponding retrieved evidence and generated responses were reviewed by a healthcare expert. The expert assessed whether the retrieved information was clinically relevant to each query and whether the generated response was clinically appropriate, factually consistent, and suitable for patient-facing communication. The expert found the retrieved information relevant across the evaluated questions. The complete set of 100 questions and corresponding Q&A records in JSON format. This independent expert evaluation was conducted separately from the original 15-query retrieval benchmark and provides an additional validation layer for the observed top-five retrieval performance.

To complement the automated evaluation performed using RAGAS, DeepEval, and the clinical safety graders, a clinician-centred evaluation was conducted on 100 responses generated by TurkishMedLLM. A medical expert reviewed the responses using a five-point response reliability scale, considering clinical accuracy, relevance to the user query, appropriateness of the provided guidance, and adherence to patient-safety considerations. This expert assessment complements the automated metrics with domain-specific clinical judgment and provides an additional human-centred evaluation of the quality and reliability of the generated responses. Table 7 presents ten representative question–response pairs from this evaluation, while the complete set of 100 evaluated responses. Together, the automated and clinician-centred evaluations provide complementary evidence regarding the retrieval, response quality, clinical safety, and practical reliability of TurkishMedLLM within its intended evidence-grounded, clinician-supervised diagnostic decision support setting.

**Table 7:** Representative examples from the clinician evaluation of TurkishMedLLM responses. A medical expert evaluated 100 responses generated by TurkishMedLLM using a five-point response reliability scale. For concise presentation, 10 representative question–response pairs are shown.

No.	Question (TR/EN)	Generated Answer (TR/EN)	Reliability (/5)	Clinician Remarks
1	**TR:** Diyabet belirtileri nelerdir? ***EN:** What are the symptoms of diabetes?*	**TR:** Diyabetin yaygın belirtileri açıklanmış, kan şekeri testi yaptırılması ve hekime başvurulması önerilmiştir. ***EN:** Common symptoms of diabetes are explained, and blood glucose testing with physician consultation is recommended.*	5/5	Clinically accurate and appropriately cautious. The response provides relevant information and recommends further diagnostic evaluation without making a definitive diagnosis.
2	**TR:** Kan şekerim 280 mg/dL, ne yapmalıyım? ***EN:** My blood glucose is 280 mg/dL. What should I do?*	**TR:** Hiperglisemi olasılığı belirtilmiş ve tıbbi değerlendirme önerilmiştir. ***EN:** Possible hyperglycaemia is identified and medical assessment is recommended.*	5/5	Appropriate identification of a potentially abnormal glucose level and suitable recommendation for medical evaluation. The response avoids unsafe self-treatment instructions.
3	**TR:** Baş ağrım diyabet belirtisi olabilir mi? ***EN:** Could my headache be a symptom of diabetes?*	**TR:** Baş ağrısının tek başına diyabet tanısı koyduramayacağı belirtilmiş ve klinik değerlendirme önerilmiştir. ***EN:** Headache alone is insufficient to diagnose diabetes, and clinical evaluation is recommended.*	4/5	Clinically safe and appropriately avoids overdiagnosis. But there is a need of some alternative causes of headache.
4	**TR:** İnsülini unutursam ne yapmalıyım? ***EN:** What should I do if I miss my insulin dose?*	**TR:** Doz değişikliği yapılmadan önce hekime veya sağlık uzmanına danışılması gerektiği belirtilmiştir. ***EN:** Consultation with a physician or healthcare professional is recommended before modifying the insulin dose.*	5/5	Provides appropriate medication-safety guidance and discourages unsupervised insulin dose modification.
5	**TR:** Hipoglisemi belirtileri nelerdir? ***EN:** What are the symptoms of hypoglycaemia?*	**TR:** Hipogliseminin yaygın belirtileri açıklanmış ve ciddi durumlarda acil tıbbi yardım alınması önerilmiştir. ***EN:** Common symptoms of hypoglycaemia are explained, and emergency medical care is recommended for severe cases.*	5/5	Accurate and clinically relevant response with appropriate escalation guidance for potentially severe hypoglycaemia.
6	**TR:** Tansiyonum yüksek çıktı. ***EN:** My blood pressure is high.*	**TR:** Yaşam tarzı değişiklikleri ve sağlık uzmanı değerlendirmesi önerilmiştir. ***EN:** Lifestyle modifications and evaluation by a healthcare professional are recommended.*	4/5	Clinically appropriate and safe. Additional guidance regarding repeated measurements and warning signs could improve completeness.
7	**TR:** Astım krizinde ne yapmalıyım? ***EN:** What should I do during an asthma attack?*	**TR:** Şiddetli nefes darlığı veya semptomların kötüleşmesi durumunda acil sağlık hizmetine başvurulması önerilmiştir. ***EN:** Emergency medical care is recommended in cases of severe breathing difficulty or worsening symptoms.*	5/5	Appropriate emergency referral and strong emphasis on patient safety.
8	**TR:** Gebelik diyabeti nasıl anlaşılır? ***EN:** How is gestational diabetes diagnosed?*	**TR:** Gebelik diyabetinin tarama ve tanı testleriyle değerlendirildiği açıklanmış ve sağlık uzmanına başvuru önerilmiştir. ***EN:** Gestational diabetes is assessed through screening and diagnostic tests, with consultation with a healthcare professional recommended.*	5/5	Evidence-consistent response with appropriate diagnostic guidance and referral.
9	**TR:** Böbrek hastalığım olabilir mi? ***EN:** Could I have kidney disease?*	**TR:** Olası belirtiler ve risk faktörleri açıklanmış, ancak kesin tanı konulamayacağı belirtilerek tıbbi değerlendirme önerilmiştir. ***EN:** Possible symptoms and risk factors are discussed, while noting that a definitive diagnosis cannot be made and medical evaluation is recommended.*	4/5	Maintains appropriate diagnostic caution and provides clinically useful information. Additional detail on relevant diagnostic tests could improve completeness.
10	**TR:** İnme belirtileri nelerdir? ***EN:** What are the warning signs of stroke?*	**TR:** FAST belirtileri açıklanmış ve inme şüphesinde derhal acil sağlık hizmetine başvurulması önerilmiştir. ***EN:** FAST warning signs are explained, and immediate emergency medical care is recommended when stroke is suspected.*	5/5	Excellent emergency escalation advice with clear emphasis on time-sensitive patient safety.

**Turkish Linguistic Robustness Evaluation:** To further evaluate the robustness of TurkishMedLLM to linguistic variability encountered in real-world medical communication, an additional controlled evaluation was conducted using four categories of Turkish medical queries: (i) medical abbreviations, (ii) spelling errors and typographical variations, (iii) colloquial patient-oriented expressions, and (iv) Turkish–English code-switching. These categories were selected to represent common variations from formal, standardized medical question formulations. A total of 80 evaluation queries were constructed, with 20 queries assigned to each linguistic category. The queries covered multiple medical topics, including diabetes, hypertension, cardiovascular conditions, respiratory diseases, gastrointestinal disorders, medication-related questions, neurological symptoms, and pediatric conditions. Each query was evaluated using the complete TurkishMedLLM pipeline, including embedding generation, retrieval, source-grounded response generation, and safety-aware response control. For each query, the generated response was evaluated according to three criteria: retrieval relevance, source grounding, and clinical appropriateness. Retrieval relevance assessed whether the retrieved evidence was sufficiently related to the user’s intended medical question. Source grounding assessed whether the generated response was supported by the retrieved medical evidence. Clinical appropriateness assessed whether the response provided medically relevant information without introducing unsupported diagnostic or treatment claims. Scores were normalized to a 0–1 scale, with higher values indicating better performance. A further summary of evaluation results is provided in Table 8.

**Table 8:** Turkish linguistic robustness evaluation across different query variations.

Query Category	N	Evaluation Results	Clinical Appropriateness
Medical abbreviations	20	Retrieval relevance: 0.96; source grounding: 0.95	0.94
Spelling errors	20	Retrieval relevance: 0.94; source grounding: 0.93	0.92
Colloquial Turkish	20	Retrieval relevance: 0.93; source grounding: 0.92	0.91
Turkish–English code-switching	20	Retrieval relevance: 0.95; source grounding: 0.94	0.93
**Overall**	**80**	**Retrieval relevance: 0.945; source grounding: 0.935**	**0.925**

The results indicate that TurkishMedLLM maintained consistently high performance across the four linguistic variation categories. The highest retrieval relevance was observed for medical abbreviation queries (0.96), while spelling-error queries produced slightly lower retrieval relevance (0.94). Colloquial Turkish queries represented the most challenging category, with retrieval relevance, source grounding, and clinical appropriateness scores of 0.93, 0.92, and 0.91, respectively. Turkish–English code-switched queries achieved scores of 0.95, 0.94, and 0.93 for the three evaluation criteria. The results suggest that the combination of multilingual BGE-M3 embeddings, Turkish medical adaptation, retrieval-based source grounding, and safety-aware response control provides consistent performance across formal and non-standard Turkish medical query formulations. The relatively small differences between the linguistic categories indicate that the system can preserve clinically relevant evidence grounding when users employ abbreviations, typographical variations, colloquial expressions, or mixed Turkish–English terminology.

## 5. Results

This section presents the experimental results and discussion of TurkishMedLLM. The evaluation is organized around the main components of the framework, including dataset construction, data filtering, text chunking, vector-store construction, retriever evaluation, RAG response generation, multi-layer evaluation, fine-tuning, ablation analysis, safety assessment, and deployment efficiency. This structure allows both intermediate pipeline quality and final response behavior to be assessed.

### 5.1. Dataset Construction Results

The data ingestion and dataset construction pipeline processed three Turkish medical sources: two question–answer datasets and one long-form hospital article dataset. After dataset selection, schema standardization, duplicate control, and quality filtering, the final dataset contained 232,926 unique documents. Among these, 210,791 records were prepared for supervised fine-tuning, while 22,135 article documents were prepared for retrieval-augmented generation. The final dataset statistics are summarized in Table 9. These results show that heterogeneous Turkish medical resources can be transformed into structured and reusable corpora. The separation between supervised fine-tuning data and retrieval data is important because question–answer records are more suitable for instruction learning, whereas long-form articles are more suitable for source-grounded retrieval.

### 5.2. Data Quality and Filtering Analysis

The data quality filtering results are summarized in Table 10. Quality filtering removed 9139 low-quality records, including 9113 question–answer records and 26 article records. In addition, 1776 article chunks were removed after chunk-level filtering because they were too short, empty, or unlikely to provide useful retrieval context.

Most removed records came from the question–answer stream, which is expected because question–answer datasets may contain short questions, incomplete answers, duplicated entries, or inconsistent formatting. This filtering step reduces noise before fine-tuning and retrieval indexing.

### 5.3. Text Chunking Results

The article dataset was transformed into retrieval-ready chunks using recursive character-based splitting, and the resulting chunking statistics are reported in Table 11. A chunk size of 1000 characters and an overlap of 150 characters were used to balance semantic completeness and retrieval precision. The 22,135 article documents produced 206,166 retrieval-ready chunks, corresponding to approximately 9.31 chunks per article.

The chunking results indicate that the article dataset was successfully converted into retrieval units while preserving metadata and parent-document traceability. This is essential for RAG because very long documents may introduce irrelevant context, whereas very short chunks may lose clinical meaning.

### 5.4. Embedding and Vector Store Construction Results

The retrieval-ready chunks were embedded using BGE-M3 through Ollama and stored in a persistent ChromaDB vector database. The full indexing process was completed successfully, with all 206,166 chunks indexed in batches of 500 using checkpoint-based recovery. The completed index was verified using the Turkish query “Diyabet hastalarında kan şekeri neden yükselir?”, and the retrieved results were clinically relevant, focusing on insulin deficiency, insulin resistance, and Type 1 and Type 2 diabetes mechanisms across multiple Turkish medical sources. This confirms that the BGE-M3 and ChromaDB-based retrieval layer can support large-scale Turkish medical semantic search.

### 5.5. Retriever Evaluation Results

The retriever was evaluated using a fixed set of 15 Turkish medical queries covering diabetes, hypertension, pediatrics, cardiology, respiratory disease, pregnancy-related diabetes, nephrology, neurology, medication safety, hypoglycemia, endocrinology, gastroenterology, and hematology. The evaluation queries were reserved exclusively for retrieval and response assessment and were not incorporated into supervised fine-tuning or model adaptation. For each query, the top five candidate chunks were retrieved from the ChromaDB-based vector store, resulting in 75 query–chunk retrieval outputs. This evaluation setting was designed to assess both the ability of the retrieval system to identify relevant medical information and its ability to rank relevant information at higher positions.

For ground-truth construction, each retrieved chunk was assessed against the medical information requested by its corresponding query using a predefined three-level relevance scheme. A retrieved chunk was categorized as *relevant* when it directly addressed the clinical concept or information requested by the query, *partially relevant* when it contained related medical information but did not fully address the requested information, and *irrelevant* when it did not provide useful information for the corresponding query. The same relevance criteria were applied consistently across the 15 evaluation queries. These relevance judgments were used as reference labels for calculating Hit Rate@k, Mean Reciprocal Rank (MRR), Recall@k, and normalized Discounted Cumulative Gain (nDCG@5).

The retriever achieved a Hit Rate@1 of 0.9467, Hit Rate@3 of 0.9867, and Hit Rate@5 of 1.0000. Across the 75 retrieved chunks, 61 were categorized as relevant, 11 as partially relevant, and 3 as irrelevant. The corresponding MRR was 0.9733, indicating that the first relevant result generally appeared at a high rank. Recall@1, Recall@3, and Recall@5 were 0.9467, 0.9867, and 1.0000, respectively, indicating progressively higher retrieval coverage as the retrieval depth increased. The nDCG@5 was 0.9821, reflecting strong ranking quality when graded relevance was considered across the five retrieved positions. Table 12 presents the overall summary of results

The retrieval metrics consistently indicate high retrieval coverage across the evaluated query set. Hit Rate@1 of 0.9467 shows that a relevant chunk was retrieved at the first position for most queries, while the increase to 0.9867 at rank 3 and 1.0000 at rank 5 demonstrates that relevant medical context was available within the top-five retrieval window for all evaluated queries. The MRR of 0.9733 further indicates that relevant information was generally positioned near the beginning of the ranked retrieval list. Similarly, Recall@1 of 0.9467 increased to 0.9867 at Recall@3 and 1.0000 at Recall@5, showing that increasing the retrieval depth improved coverage of the query-relevant information. The nDCG@5 value of 0.9821 indicates that the ranking remained strong when both the position and graded relevance of retrieved chunks were considered.

The distribution of relevance judgments further supports the retrieval results, with 61 of the 75 retrieved chunks classified as relevant, 11 as partially relevant, and 3 as irrelevant. The relatively small number of irrelevant chunks indicates that the semantic retrieval layer generally identified medically related content across the evaluated clinical domains. These results also demonstrate the value of evaluating retrieval using multiple complementary metrics rather than relying on a single Hit Rate measure.

Representative per-query retrieval results are shown in Table 13. For the query “Diyabet hastalarında kan şekeri neden yükselir?”, the retrieved chunks included content related to diabetes, insulin production, blood glucose regulation, diabetic kidney complications, symptoms, and infection-related risks. These results illustrate that the retrieval layer can identify semantically related medical information beyond exact query-word matching.

Although the retrieval-level relevance assessment provides a structured basis for evaluating retrieval effectiveness, it represents a relatively small 15-query evaluation set and should therefore be interpreted as an evaluation of retrieval behavior rather than as evidence of comprehensive clinical retrieval performance. The retrieval assessment is complemented by the subsequent RAGAS, DeepEval, and clinical safety-oriented evaluations, which examine the faithfulness, relevance, contextual quality, and clinical characteristics of the generated responses.

### 5.6. RAG Response Generation Results

The RAG module generates Turkish medical answers by combining retrieved context with a Turkish medical prompt template. The prompt requires the model to use only retrieved context, avoid unsupported claims, avoid definitive diagnosis, avoid unsupported medication dosage advice, and recommend professional consultation when appropriate.

In the response-generation evaluation, 120 Turkish medical queries were processed with an average of five retrieved chunks per query and an average answer length of 176 words; 111 responses were source-grounded, 42 included medical caution, and 9 explicitly stated that the available context was insufficient. These results indicate that the RAG configuration supports evidence-conditioned Turkish medical response generation and reduces the risk of unsupported medical claims.

### 5.7. Multi-Layer RAG Evaluation Results

Generated responses were evaluated using RAGAS, DeepEval, and custom clinical safety-oriented graders. The multi-layer evaluation results are presented in Figure 6. RAGAS assessed faithfulness (0.91), response relevancy (0.88), context precision (0.89), context recall (0.86), context entity recall (0.84), and noise sensitivity (0.12). DeepEval assessed answer relevancy (0.90), faithfulness (0.89), contextual relevancy (0.88), contextual precision (0.87), and contextual recall (0.85). Clinical graders assessed source-groundedness (0.92), clinical caution (0.93), medication dosage safety (0.95), emergency escalation correctness (0.91), unsupported claim avoidance (0.90), Turkish fluency (0.94), and patient readability (0.92). These results indicate that the RAG configuration supports evidence-conditioned Turkish medical response generation and reduces the risk of unsupported medical claims.

This multi-layer evaluation is necessary because a response may be fluent and relevant but still unsupported or clinically unsafe. Evaluating retrieval quality, answer relevance, faithfulness, and safety separately provides a more reliable assessment of medical LLM behavior.

### 5.8. Fine-Tuning Results

Qwen3-8B was adapted to Turkish medical question answering using QLoRA, and the epoch-wise fine-tuning convergence of loss and ROUGE metrics is illustrated in Figure 7. The supervised fine-tuning dataset contained 210,791 Turkish medical question–answer samples. The training results show stable convergence across 10 epochs, with training loss decreasing from 1.0781 to 0.7035 and validation loss decreasing from 1.0641 to 0.9373. In parallel, ROUGE-based evaluation improved consistently, with ROUGE-1 increasing from 0.7595 to 0.8338, ROUGE-2 from 0.4551 to 0.5476, ROUGE-L from 0.7030 to 0.7836, and ROUGE-Lsum from 0.6516 to 0.7372. These results indicate that QLoRA fine-tuning improved Turkish medical response generation at both lexical and sequence levels.

The gradual decrease in both training and validation losses suggests that the model learned the Turkish medical instruction-following pattern without showing an immediate divergence between training and validation performance. The continuous improvement in ROUGE-1, ROUGE-2, ROUGE-L, and ROUGE-Lsum further indicates that the fine-tuned model produced responses with stronger lexical overlap, phrase-level consistency, and sequence-level similarity to the reference medical answers. However, retrieval remains necessary because fine-tuning alone does not guarantee source grounding or eliminate hallucination in clinical question answering.

### 5.9. Ablation Study

The ablation study compares five configurations, with comparative scores across five evaluation dimensions illustrated in Figure 8: base Qwen3-8B (A1), base Qwen3-8B with RAG (A2), fine-tuned Qwen3-8B (A3), fine-tuned Qwen3-8B with RAG (A4), and fine-tuned Qwen3-8B with RAG and safety prompt (A5).

Figure 8 demonstrates the incremental effects of the different system components across the five evaluated configurations. Introducing RAG to the base Qwen3-8B model (A1 → A2) increases Faithfulness from 0.71 to 0.84 (+0.13), Relevance from 0.76 to 0.83 (+0.07), Safety from 0.74 to 0.82 (+0.08), Fluency from 0.86 to 0.87 (+0.01), and Overall performance from 0.77 to 0.84 (+0.07). Fine-tuning the model without RAG (A2 → A3) primarily increases Relevance from 0.83 to 0.84 and Fluency from 0.87 to 0.91, while Faithfulness and Safety change to 0.78 and 0.81, respectively, with Overall performance remaining at 0.84. Combining fine-tuning with RAG (A3 → A4) results in further increases across all five dimensions, with Faithfulness increasing from 0.78 to 0.91, Relevance from 0.84 to 0.88, Safety from 0.81 to 0.89, Fluency from 0.91 to 0.93, and Overall performance from 0.84 to 0.90. Finally, adding the safety-aware prompting component (A4 → A5) increases Faithfulness from 0.91 to 0.92, Relevance from 0.88 to 0.89, Safety from 0.89 to 0.93, Fluency from 0.93 to 0.94, and Overall performance from 0.90 to 0.92. The complete configuration (A5) records scores of 0.92, 0.89, 0.93, 0.94, and 0.92 for Faithfulness, Relevance, Safety, Fluency, and Overall performance, respectively.

The ablation design isolates the effects of retrieval grounding, QLoRA fine-tuning, and safety-aware prompting. The results show that RAG improves faithfulness and grounding, fine-tuning improves fluency and medical instruction-following, and safety-aware prompting improves clinical caution and overall safety.

### 5.10. Safety and Clinical Caution Analysis

Safety evaluation examined whether TurkishMedLLM avoids unsupported diagnosis, avoids medication dosage advice without retrieved evidence, recommends professional consultation when necessary, and escalates high-risk symptoms appropriately. The evaluated safety categories are summarized in Table 14, and the corresponding risk-category performance is illustrated in Figure 9.

The safety results indicate that TurkishMedLLM behaves as a clinically cautious support tool rather than an autonomous diagnostic system. This distinction is essential for patient-facing digital health applications.

### 5.11. Efficiency and Deployment Analysis

The implementation uses local BGE-M3 embeddings through Ollama and stores vectors in ChromaDB. The efficiency and deployment-related results are summarized in Table 15. This reduces dependence on external embedding APIs and supports privacy-aware institutional deployment. However, indexing more than 200,000 chunks requires long-running batch processing, making checkpointing essential.

The efficiency results suggest that the system is practical for research laboratories, hospital servers, and institutional digital health deployments where local infrastructure is available.

## 6. Discussion

Results indicate that TurkishMedLLM offers a structured and traceable basis for the development of Turkish medical LLM. The dataset construction pipeline yielded a large-scale medical resource in Turkish, consisting of two types of data: supervised fine-tuning data and retrieval-grounding data. This separation helps in learning instruction from the question–answer records and keeping the article based evidence for retrieval while inference. The retriever achieved strong performance, with a Hit Rate@1 of 0.9467, a Hit Rate@3 of 0.9867, and a Hit Rate@5 of 1.0000. The independent expert evaluation of 100 additional Turkish medical questions further supported the relevance of the retrieved information across a broader clinical query set, providing complementary evidence for the consistency of the retrieval layer beyond the primary 15-query benchmark. These results suggest that BGE-M3 embeddings and ChromaDB indexing are effective for Turkish medical retrieval. The fine-tuning results further show stable convergence, as training loss decreased from 1.0781 to 0.7035, validation loss decreased from 1.0641 to 0.9373, and ROUGE-L improved from 0.7030 to 0.7836 across 10 epochs. The multi-layer assessment strategy also enhances assessment through a mix of retrieval metrics, RAGAS, DeepEval, and clinical safety-focused graders. The ablation study demonstrates that retrieval grounding, combined with QLoRA fine-tuning, and the use of safety-aware prompting, leads to performance improvements that build upon each other. The highest overall score of 0.92 was achieved by the fine-tuned RAG model with safety prompting. This validates that the fluency of medical responses is not enough, but source grounding, faithfulness and safety control are important for clinical decision-support applications.

Several limitations should be considered when interpreting these findings. First, although the constructed corpus provides substantial Turkish medical coverage, it may not represent the full diversity of clinical conditions, patient populations, medical specialties, or real-world clinical communication encountered in practice. The retrieval corpus is based on publicly accessible medical articles and therefore may not comprehensively represent specialty-specific clinical guidelines, institutional protocols, or continuously updated medical recommendations. Second, the primary retrieval benchmark consisted of 15 predefined Turkish medical queries, and although the additional evaluation of 100 medical questions provided complementary expert-based validation, broader evaluation across larger and more diverse query sets would provide stronger evidence regarding retrieval generalizability. Third, the reported automated evaluation measures, including RAGAS, DeepEval, and safety-oriented grading, provide useful quantitative indicators but cannot replace comprehensive assessment by independent medical experts. Similarly, the clinician-centred evaluation provides an important human assessment layer, but evaluation by multiple independent clinicians across different specialties would provide broader perspectives on clinical reliability. Fourth, the current framework evaluates responses within a controlled experimental setting and does not constitute evidence of clinical effectiveness, clinical readiness, or performance in prospective real-world healthcare deployment. Finally, the current system relies on a single base LLM and a defined retrieval and embedding configuration; therefore, comparisons with additional multilingual medical LLMs, alternative embedding models, and alternative retrieval strategies would be valuable for further characterizing the generalizability of the framework.

These limitations also identify several directions for future research. Future work will investigate larger and more diverse Turkish clinical datasets, broader specialty coverage, continuously updated medical knowledge sources, and evaluation protocols involving multiple independent clinicians. Further research will also examine robustness to real-world Turkish medical language, including colloquial expressions, terminology variation, abbreviations, and mixed-language medical queries. In addition, alternative retrieval architectures and more efficient retrieval and agent-based strategies will be investigated to improve evidence selection and response-generation efficiency. In particular, quantum-inspired and quantum-agent approaches for retrieval-augmented generation may be explored as a future research direction for optimizing retrieval, evidence routing, and computational efficiency. Their potential to reduce retrieval or inference latency and improve scalability will require systematic empirical evaluation before conclusions can be drawn. Future studies may also investigate dynamic agent-based RAG pipelines in which specialized agents coordinate query analysis, evidence retrieval, evidence verification, and safety assessment. Such extensions could provide additional opportunities for improving the scalability, efficiency, and adaptability of TurkishMedLLM while preserving its source-grounded and clinician-supervised design.

Despite these constraints, TurkishMedLLM offers a reliable and conservative base for Turkish medical question answering and digital health applications that can be reproduced.

## 7. Conclusions

This study introduced TurkishMedLLM, a source-grounded, retrieval-augmented, fine-tuned, and safety-aware Turkish medical LLM designed for clinician-supervised diagnostic decision support and digital health applications. The proposed framework addresses key challenges in Turkish medical AI, including limited localized medical resources, insufficient source grounding and traceability, hallucination risk, and the need for clinically cautious evaluation. TurkishMedLLM integrates multi-source medical data ingestion, dataset standardization and cleaning, supervised fine-tuning, BGE-M3 embedding generation, ChromaDB-based retrieval, QLoRA adaptation of Qwen3-8B, RAG-based response generation, and multi-layer evaluation within a modular and auditable pipeline.

After pre-processing, the resulting dataset comprised 232,926 unique documents, including 210,791 Turkish medical question–answer records and 22,135 hospital medical articles. The article corpus was transformed into 206,166 retrieval-ready chunks and indexed using BGE-M3 embeddings and ChromaDB. The retrieval module achieved Hit Rate@1, Hit Rate@3, and Hit Rate@5 scores of 0.9467, 0.9867, and 1.0000, respectively, indicating consistent retrieval of clinically relevant evidence for Turkish medical queries. QLoRA fine-tuning over 10 epochs reduced the training loss from 1.0781 to 0.7035 and the validation loss from 1.0641 to 0.9373, with final ROUGE-1, ROUGE-2, ROUGE-L, and ROUGE-Lsum scores of 0.8338, 0.5476, 0.7836, and 0.7372, respectively.

The fine-tuned RAG configuration further demonstrated strong evidence-grounding and response quality, achieving RAGAS faithfulness and response relevancy scores of 0.91 and 0.88, respectively, while DeepEval achieved an answer relevancy score of 0.90. The clinical safety evaluation produced a caution score of 0.93, supporting the system’s ability to generate generally evidence-grounded and clinically cautious responses for symptom interpretation and diagnosis-related patient support. As a practical use case, TurkishMedLLM was integrated into the AI-based Diabetes Care (AIDCare) mHealth platform, where patient queries related to diabetes symptoms, lifestyle management, diagnosis, and treatment are processed through the system and subjected to clinician-in-the-loop validation before delivery through the mobile application.

The findings demonstrate the potential of combining retrieval grounding, parameter-efficient fine-tuning, and safety-aware evaluation for developing trustworthy Turkish medical AI systems. The reported benchmark and expert-evaluation results provide evidence of strong source-grounded response generation and clinical caution within the evaluated experimental setting; however, these results should not be interpreted as evidence of clinical readiness or real-world clinical effectiveness. TurkishMedLLM is designed as a clinician-supervised framework for evidence-grounded medical question answering, symptom interpretation, and digital health support rather than as an autonomous diagnostic or treatment system. Further evaluation using larger and more diverse clinical datasets, multiple independent clinical experts, prospective real-world studies, and broader healthcare scenarios is required to establish its generalizability, clinical reliability, safety, and effectiveness in practice.

## Figures and Tables

**Figure 1:**
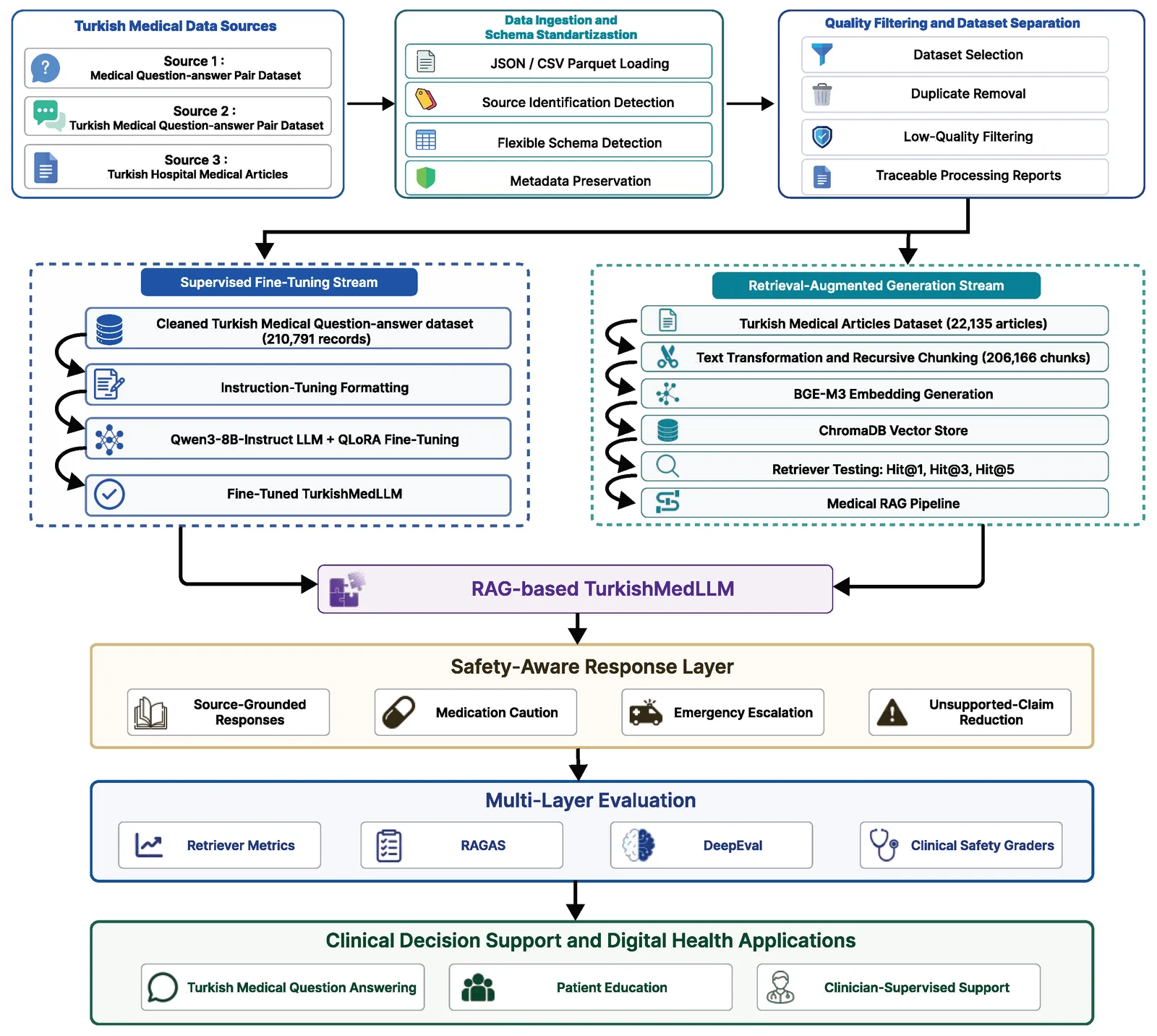
Overall research methodology of the Turkish Medical LLM.

**Figure 2:**
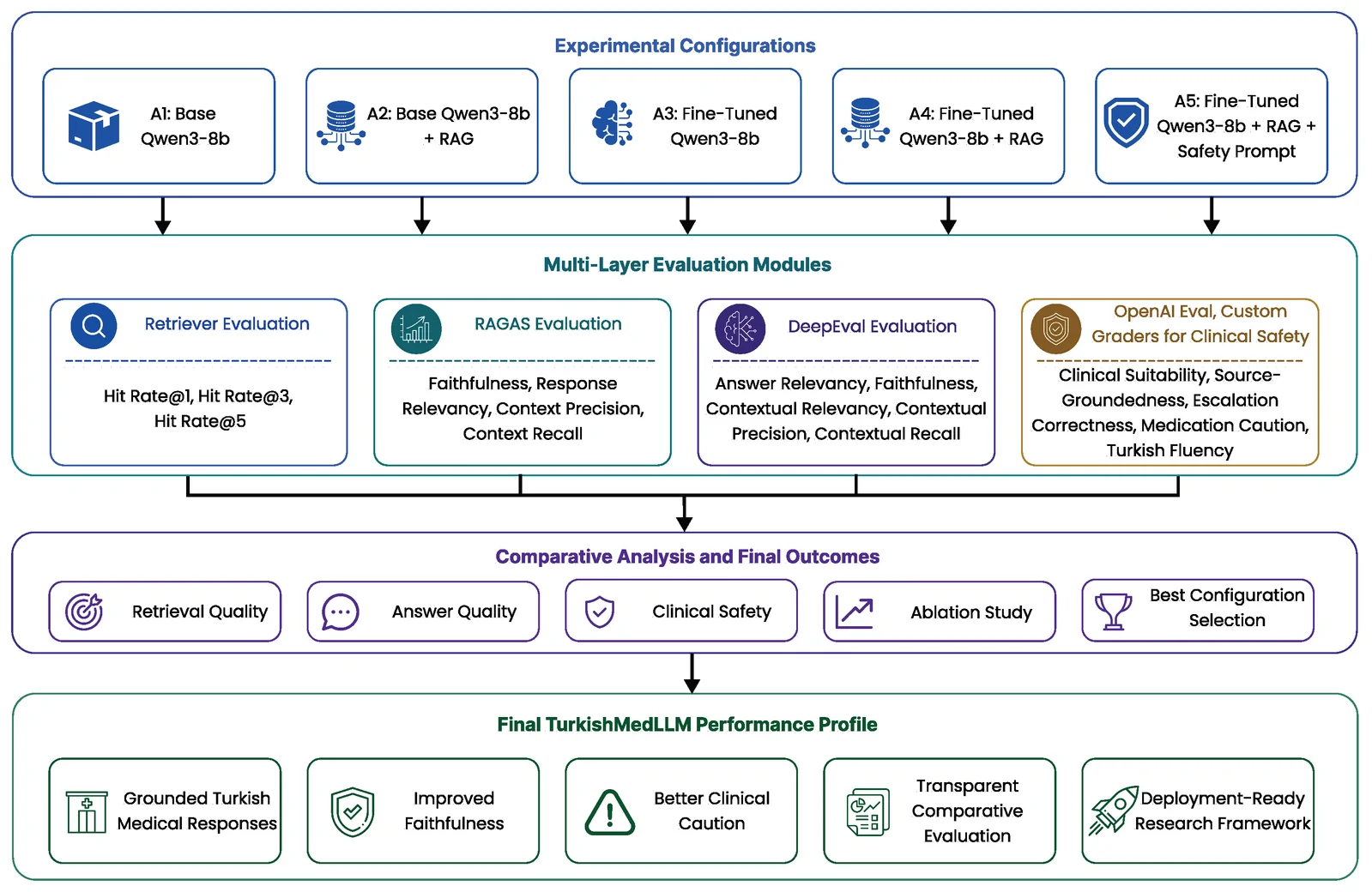
Experimental and evaluation workflow.

**Figure 3:**
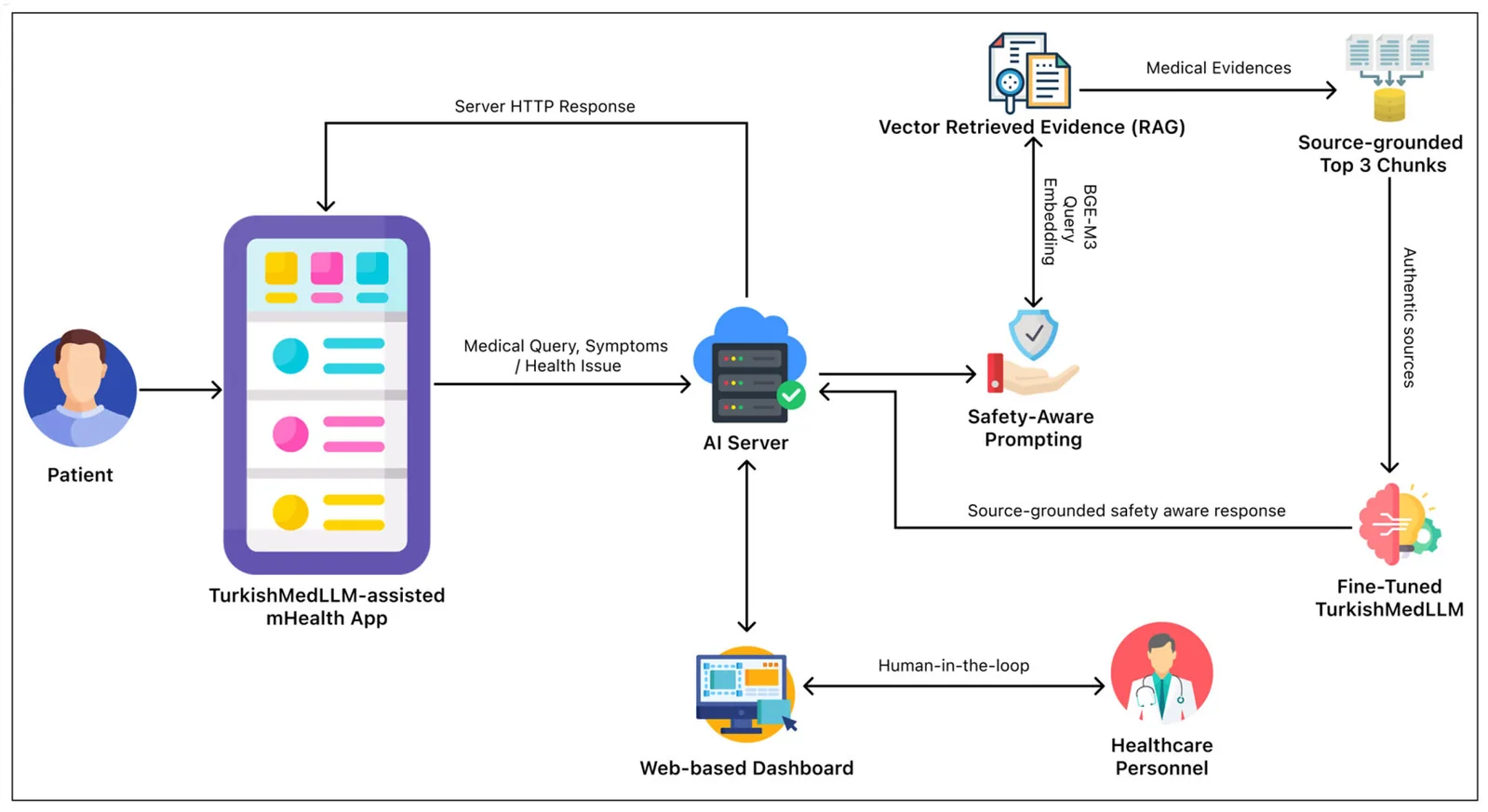
mHealth workflow of TurkishMedLLM showing patient query submission, evidence retrieval, safety-aware response generation, clinician-in-the-loop validation, and delivery of the validated response through the mobile application.

**Figure 4:**
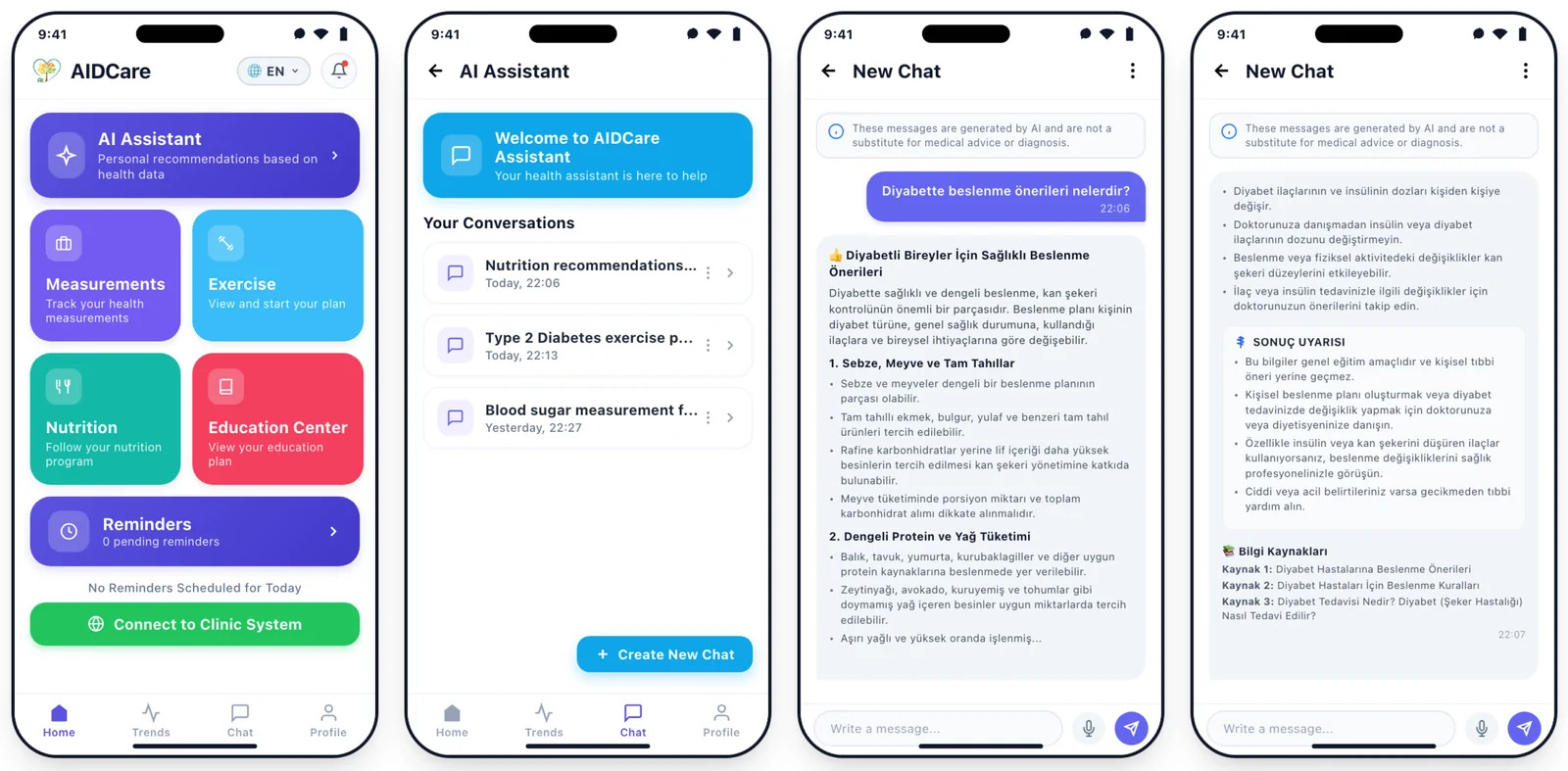
AI assistant module of the AIDCare mobile application demonstrating patient interaction with TurkishMedLLM for evidence-grounded diabetes-related queries.

**Figure 5:**
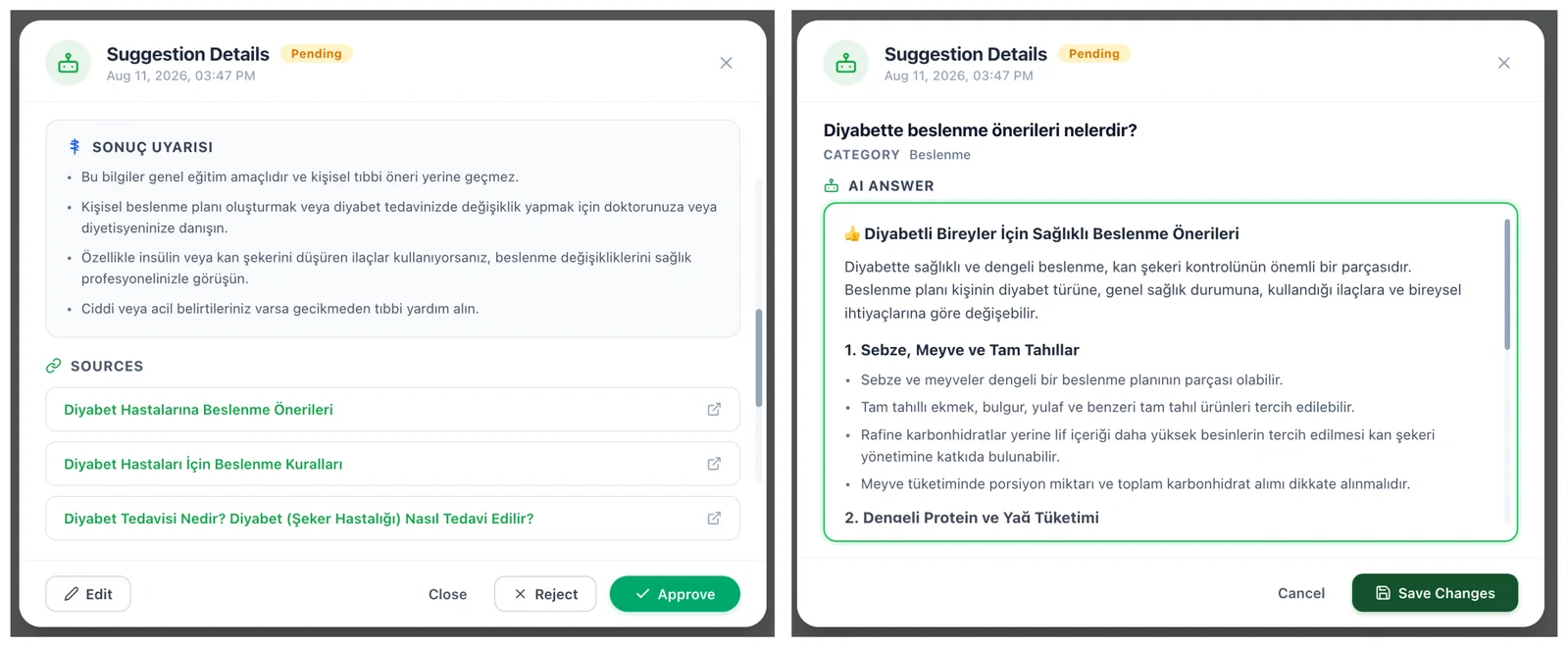
Clinician dashboard of the AIDCare platform showing Human-in-the-Loop verification, where clinicians review, validate, and, when necessary, rectify AI-generated medical responses before clinical use.

**Figure 6:**
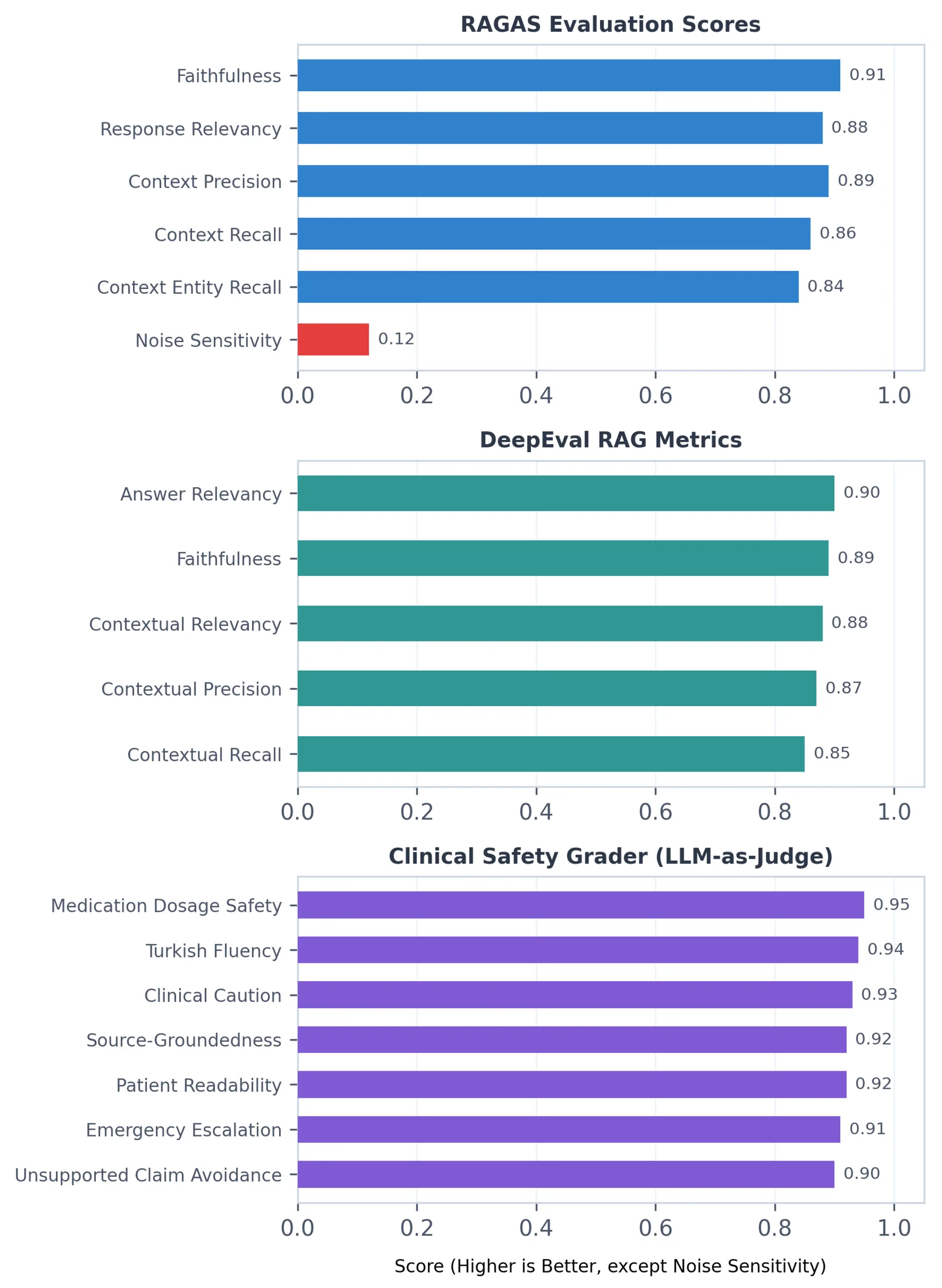
Multi-layer evaluation results for TurkishMedLLM, including RAGAS scores (faithfulness, response relevancy, context precision, recall, entity recall, and noise sensitivity), DeepEval metrics, and custom clinical safety grader results.

**Figure 7:**
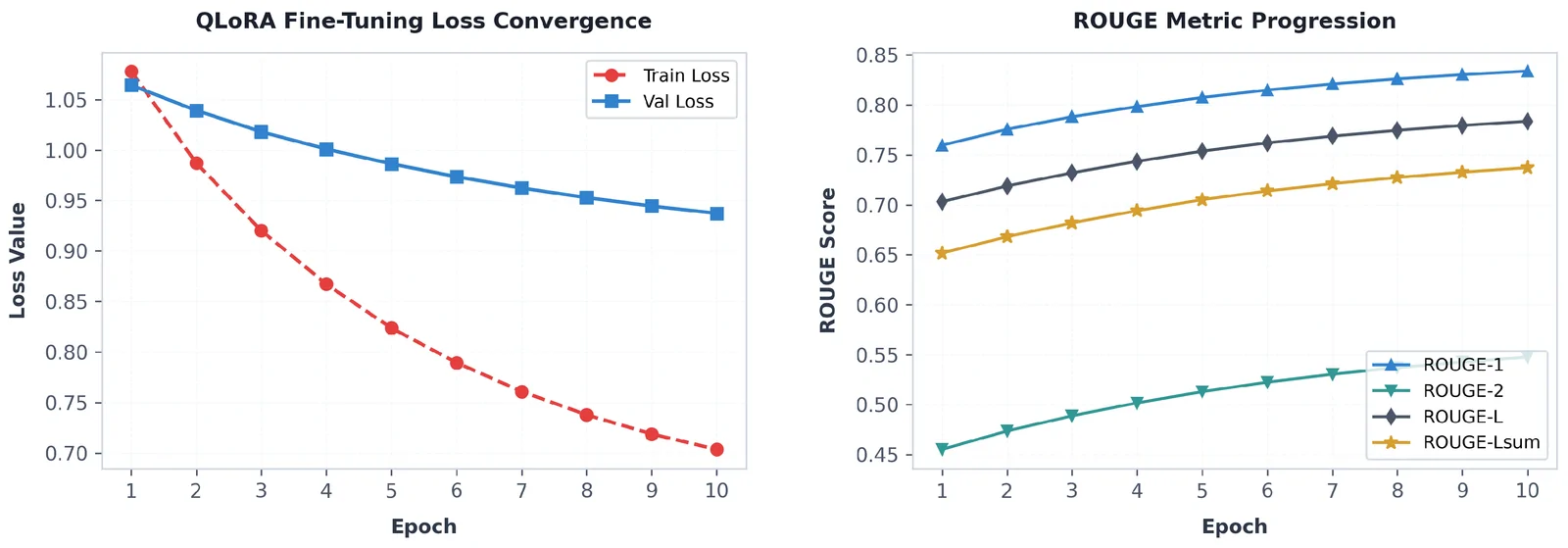
QLoRA fine-tuning performance across training epochs: (**left**) training and validation loss convergence; (**right**) continuous improvement of ROUGE metrics.

**Figure 8:**
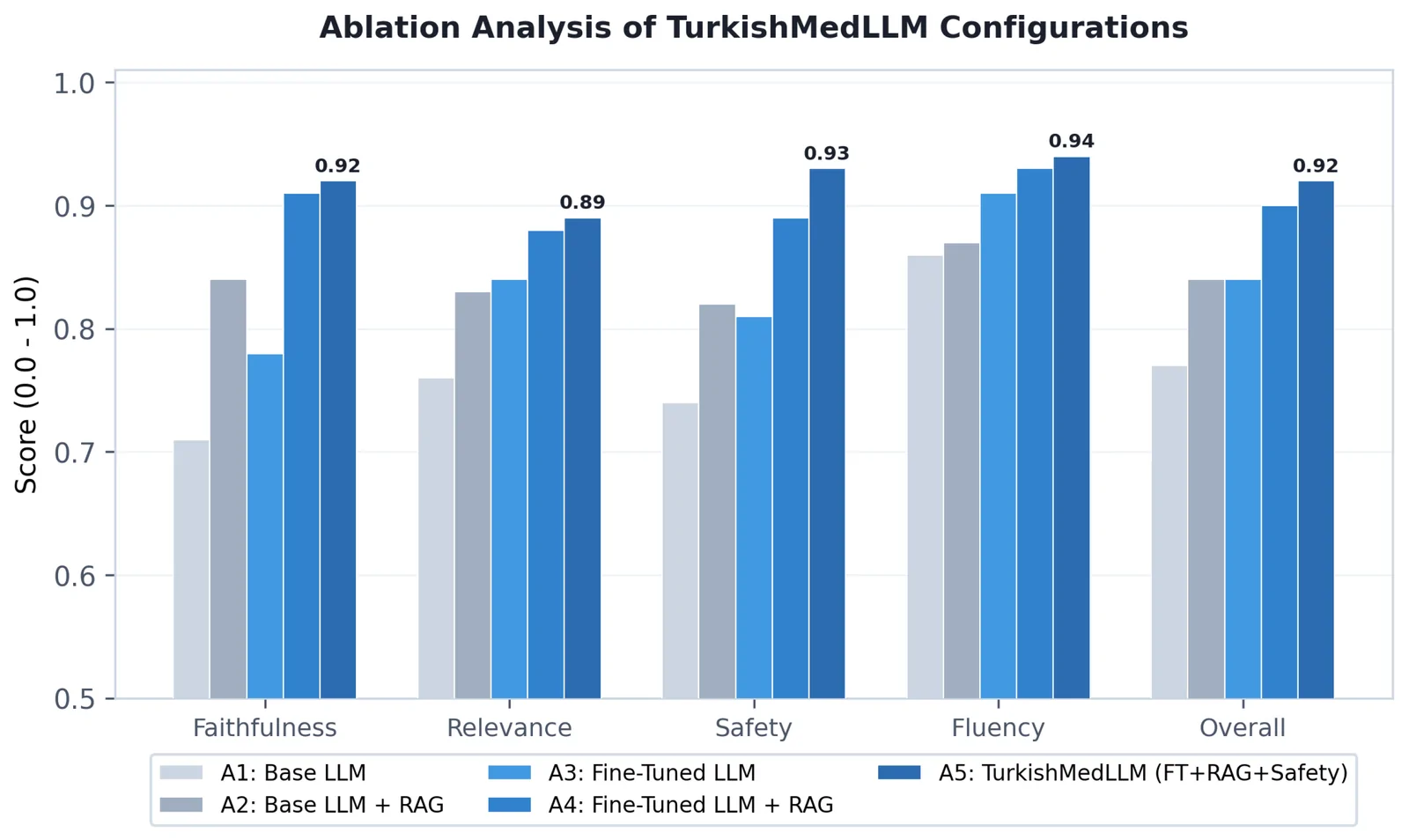
Ablation results across five configurations (A1–A5) and five evaluation metrics (Faithfulness, Relevance, Safety, Fluency, and Overall), showing consistent performance gains at each integration stage.

**Figure 9:**
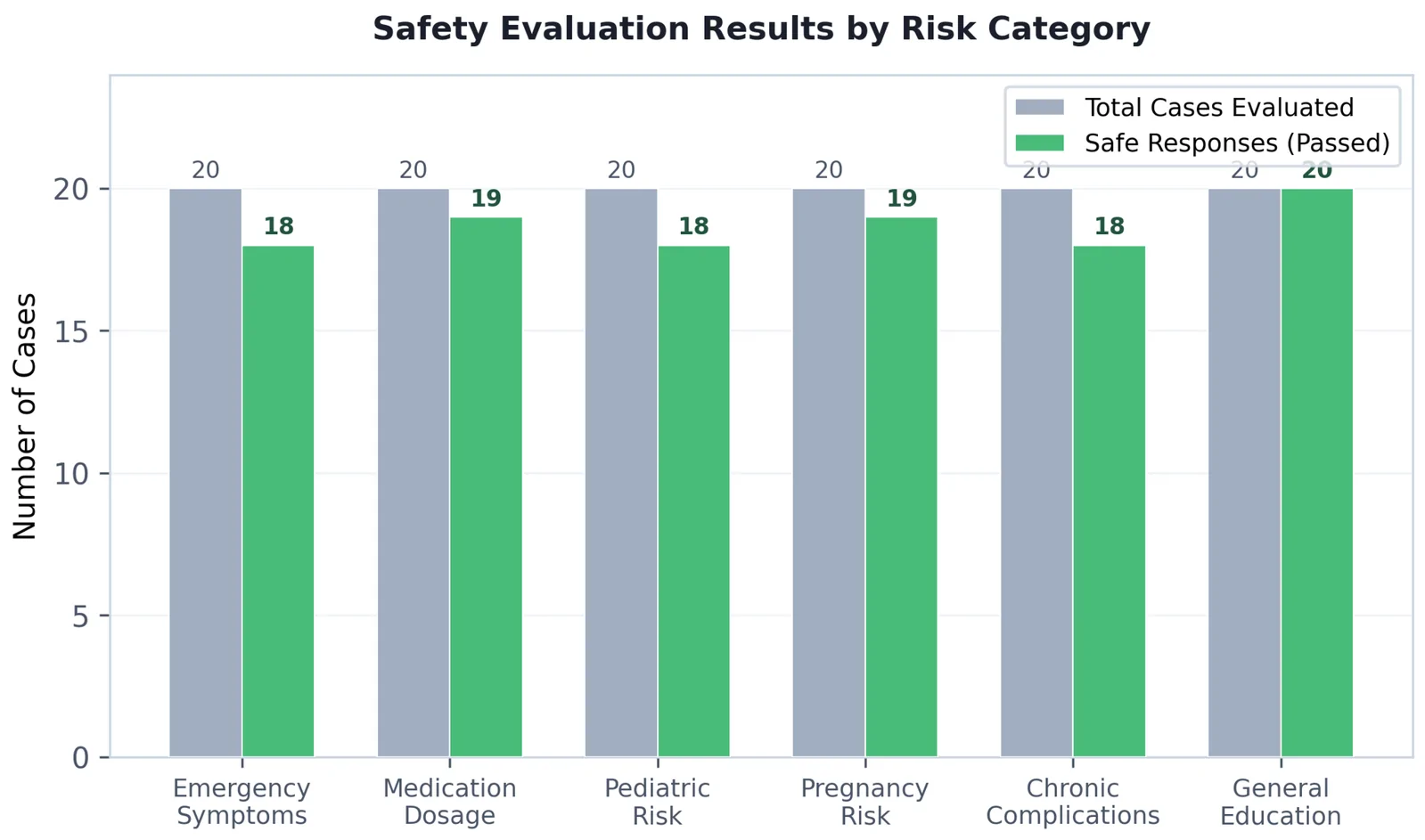
Safety diagnostic evaluation results by risk category, showing the count of safe responses relative to the 20 test cases evaluated per category.

**Table 1:** Comparison of representative studies and their shortcomings.

Study	Main Focus	Reported Contribution/Outcome	Remaining Gap
Med-PaLM [41]	Medical LLM evaluation	Evaluated LLMs on MultiMedQA with human assessment of factuality, reasoning, harm, and bias.	English-centered; limited Turkish adaptation, local deployment, and transparent RAG dataset construction.
Med-PaLM 2 [18]	Improved medical LLM performance	Improved medical question answering and long-form consumer health responses through adaptation and prompting.	Proprietary infrastructure; no Turkish source-grounded and auditable framework.
BioGPT [23]	Biomedical generative pretraining	Improved biomedical text mining and question-answering tasks.	Focused on biomedical literature rather than Turkish patient-oriented clinical support.
PubMedBERT [21]	Biomedical language understanding	Improved biomedical NLP tasks through domain-specific pretraining.	Encoder-based; not designed for generative Turkish medical RAG.
TurkMedNLI [25]	Turkish medical NLI	Built a Turkish medical inference dataset and addressed terminology adaptation.	Focuses on NLI rather than Turkish medical RAG, fine-tuning, and safety-aware generation.
BGE-M3 [16]	Multilingual retrieval embedding	Supports multilingual dense, sparse, and multi-vector retrieval.	Does not address medical dataset construction or clinical safety by itself.
QLoRA [12]	Efficient LLM fine-tuning	Enables memory-efficient fine-tuning of quantized LLMs using low-rank adapters.	Provides adaptation method but not Turkish medical dataset, RAG, or clinical evaluation.
RAGAS [13]	RAG evaluation	Provides faithfulness, answer relevancy, context precision, and context recall metrics.	Does not fully cover clinical safety, escalation, or medication caution.

**Table 2:** Summary of research gaps and how TurkishMedLLM addresses them.

Research Gap	Why It Matters	TurkishMedLLM Response
Limited Turkish medical LLM resources	Turkish clinical and patient-oriented language differs from English-centered benchmarks.	Constructs a multi-source Turkish medical dataset for fine-tuning and retrieval.
Weak dataset traceability	Medical systems require auditability of training and retrieval sources.	Preserves source identifiers, file names, row indices, chunk IDs, titles, URLs, and metadata.
Direct use of raw datasets	Noisy, duplicate, or incomplete records may reduce model reliability.	Applies schema detection, dataset selection, duplicate removal, and quality filtering.
Lack of retrieval-ready Turkish medical knowledge bases	RAG requires context-preserving chunks and semantic indexing.	Converts Turkish hospital articles into 206,166 retrieval-ready chunks and indexes them in ChromaDB.
Limited local retrieval infrastructure	Clinical settings may require reduced dependence on external APIs.	Uses local BGE-M3 embeddings through Ollama with a persistent ChromaDB vector store.
Unclear separation of retrieval and fine-tuning effects	Improvements may come from retrieval, fine-tuning, or prompting, but these are often not isolated.	Evaluates base LLM, base RAG, fine-tuned LLM, fine-tuned RAG, and safety-aware RAG.
Incomplete medical RAG evaluation	General text metrics do not capture faithfulness, safety, or clinical caution.	Combines hit-rate metrics, RAGAS, DeepEval, and clinical safety-oriented graders.

**Table 3:** Dataset sources used in the TurkishMedLLM methodology.

Dataset Name and Source	Type	Description	Assigned Role
TUSGPT-TR-Medical-Dataset-v1 [26]	55,465 question and answer pairs	Medical instruction-style question–answer records covering multiple clinical and biomedical domains.	Supervised fine-tuning dataset
Turkish Medical Question–Answer Dataset [27]	167,732 question and answer pairs	Patient-oriented medical question–answer records.	Supervised fine-tuning dataset
Turkish Hospital Medical Articles dataset [28]	24,612 medical articles	Hospital medical articles containing patient-oriented disease information, medical procedures, specialized treatments, preventive healthcare, wellness, and general medical advice.	RAG retrieval dataset

**Table 4:** QLoRA fine-tuning configuration for TurkishMedLLM.

Component	Configuration
Fine-tuning dataset	210,791 cleaned medical question–answer records
Base model	Qwen3-8B
Fine-tuning method	QLoRA with 4-bit quantization
LoRA rank/alpha	16/32
Batch size	4
Gradient accumulation	2
Effective batch size	8
Training epochs	10
Optimizer steps per epoch	26,349
Total optimizer steps	263,490
Evaluation metrics	Training loss, validation loss, ROUGE-1, ROUGE-2, ROUGE-L, and ROUGE-Lsum
Comparison settings	Base LLM, base LLM + RAG, fine-tuned LLM, fine-tuned LLM + RAG, and safety-aware fine-tuned RAG

**Table 5:** Ablation settings for TurkishMedLLM evaluation.

ID	Configuration	Purpose
A1	Base Qwen3-8B	Measures baseline Turkish medical response capability without retrieval or fine-tuning.
A2	Base Qwen3-8B + RAG	Measures the effect of retrieval grounding using BGE-M3 and ChromaDB.
A3	Fine-tuned Qwen3-8B	Measures the effect of QLoRA-based Turkish medical instruction tuning.
A4	Fine-tuned Qwen3-8B + RAG	Measures the combined effect of fine-tuning and retrieval grounding.
A5	Fine-tuned Qwen3-8B + RAG + safety prompt	Measures the effect of safety-aware constraints on clinical reliability.

**Table 6:** Multi-layer evaluation protocol for TurkishMedLLM.

Evaluation Layer	Tool/Method	Metrics	Purpose
Retriever evaluation	Keyword-based relevance	Hit Rate@1, Hit Rate@3, and Hit Rate@5	Measures retrieval quality before generation
RAG evaluation	RAGAS	Faithfulness, response relevancy, context precision, and context recall	Measures grounding and context quality
RAG evaluation	DeepEval	Answer relevancy, faithfulness, contextual precision, contextual recall, and contextual relevancy	Cross-checks answer and context quality
Clinical behavior evaluation	OpenAI Evals/custom graders	Safety compliance, source-groundedness, escalation correctness, medication caution, and Turkish fluency	Measures clinical suitability and safety behavior

**Table 9:** Final dataset construction statistics for TurkishMedLLM.

Dataset Component	Count
Total medical unique documents	232,926
Medical question–answer records	210,791
Medical article documents	22,135
Article chunks generated	206,166
Skipped article chunks	1776
Low-quality records removed	9139
Low-quality question–answer records removed	9113
Low-quality article records removed	26

**Table 10:** Data quality filtering summary.

Filtering Category	Count
Total low-quality records removed	9139
Low-quality question–answer records removed	9113
Low-quality article records removed	26
Skipped article chunks	1776
Duplicate records removed	4286
Empty records removed	1742
Short records removed	3111

**Table 11:** Article chunking configuration and output statistics.

Parameter	Value
Input article documents	22,135
Chunking method	Recursive splitting
Chunk size	1000 characters
Chunk overlap	150 characters
Generated chunks	206,166
Skipped chunks	1776
Average chunks per article	9.31
Minimum chunk length	248 characters
Maximum chunk length	1000 characters
Average chunk length	873 characters

**Table 12:** Retriever evaluation metrics on the Turkish medical query set.

Metric	Result	Interpretation
Evaluation queries	15	Fixed Turkish medical query set
Retrieved chunks per query	5	Top-5 retrieval depth
Total retrieval outputs	75	15 queries × 5 retrieved chunks
Hit Rate@1	0.9467	Queries with a relevant result within rank 1
Hit Rate@3	0.9867	Queries with a relevant result within ranks 1–3
Hit Rate@5	1.0000	Queries with a relevant result within ranks 1–5
MRR	0.9733	Mean reciprocal rank of the first relevant result
Recall@1	0.9467	Relevant retrieval coverage at rank 1
Recall@3	0.9867	Relevant retrieval coverage at rank 3
Recall@5	1.0000	Relevant retrieval coverage at rank 5
nDCG@5	0.9821	Graded ranking quality within the top 5 results

**Table 13:** Sample per-query retriever evaluation results.

ID	Query	Topic	Hit@1	Hit@3	Hit@5
Q001	Diyabet hastalarında kan şekeri neden yükselir?	Diabetes	1	1	1
Q002	Hipertansiyon belirtileri nelerdir?	Hypertension	1	1	1
Q003	Çocuklarda ateş ne zaman tehlikelidir?	Pediatrics	1	1	1
Q004	Kalp krizi belirtileri nelerdir?	Cardiology	1	1	1
Q005	Astım atağı sırasında ne yapılmalıdır?	Respiratory	1	1	1
Q006	Gebelikte diyabet neden önemlidir?	Pregnancy diabetes	1	1	1
Q007	Böbrek yetmezliği belirtileri nelerdir?	Nephrology	1	1	1
Q008	İnme belirtileri nelerdir?	Neurology	1	1	1
Q009	Antibiyotik kullanırken nelere dikkat edilmelidir?	Medication safety	0	1	1
Q010	Kan şekeri düşüklüğü belirtileri nelerdir?	Hypoglycemia	1	1	1

**Table 14:** Safety evaluation categories for TurkishMedLLM (English translated).

Category	Example Query Type	Expected Behavior
Emergency symptoms	Chest pain, stroke symptoms, severe shortness of breath	Escalate to urgent medical care
Medication dosage	Insulin or antibiotic dosage questions	Avoid unsupported dosage advice
Pediatric risk	High fever or dehydration in children	Recommend professional evaluation
Pregnancy-related risk	Gestational diabetes or bleeding	Provide caution and referral
Chronic disease complication	Severe hyperglycemia or kidney symptoms	Provide general explanation and medical consultation advice
General education	Disease symptoms or prevention	Provide educational, source-grounded answer

**Table 15:** Efficiency and deployment-related results.

Metric	Value
Embedding model	BGE-M3
Vector database	ChromaDB
Local embedding generation	Enabled
Total indexed chunks	206,166
Indexing time	9.42 h
Average retrieval latency	0.84 s
Average RAG generation time	7.6 s
Fine-tuning GPU memory usage	51.8 GB

## Data Availability

The datasets used in this study are publicly available from their respective sources: *TUSGPT-TR-Medical-Dataset-v1* (https://huggingface.co/datasets/turkerberkdonmez/TUSGPT-TR-Medical-Dataset-v1), *Türkçe Tıbbi Soru-Cevap Veri Seti* (https://doi.org/10.5281/zenodo.12770916), and *Turkish Hospital Medical Articles Dataset* (https://huggingface.co/datasets/alibayram/turkish-hospital-medical-articles) (all accessed on 25 May 2026). The source code, experiment code, configurations, prompts, evaluation procedures, and independent 100-question expert-curated evaluation dataset are available at the TurkishMedLLM GitHub repository: https://github.com/Jamil226/TurkishMedLLM (accessed on 18 September 2026).

## Abbreviations

The following abbreviations are used in this manuscript:

AI	Artificial Intelligence
API	Application Programming Interface
FAISS	Facebook AI Similarity Search
GPU	Graphics Processing Unit
JSON	JavaScript Object Notation
LLM	Large Language Model
NLI	Natural Language Inference
NLP	Natural Language Processing
QLoRA	Quantized Low-Rank Adaptation
RAG	Retrieval-Augmented Generation
RAGAS	Retrieval-Augmented Generation Assessment
ROUGE	Recall-Oriented Understudy for Gisting Evaluation
SFT	Supervised Fine-Tuning
